# Sarcoma treatment in the era of molecular medicine

**DOI:** 10.15252/emmm.201911131

**Published:** 2020-10-13

**Authors:** Thomas GP Grünewald, Marta Alonso, Sofia Avnet, Ana Banito, Stefan Burdach, Florencia Cidre‐Aranaz, Gemma Di Pompo, Martin Distel, Heathcliff Dorado‐Garcia, Javier Garcia‐Castro, Laura González‐González, Agamemnon E Grigoriadis, Merve Kasan, Christian Koelsche, Manuela Krumbholz, Fernando Lecanda, Silvia Lemma, Dario L Longo, Claudia Madrigal‐Esquivel, Álvaro Morales‐Molina, Julian Musa, Shunya Ohmura, Benjamin Ory, Miguel Pereira‐Silva, Francesca Perut, Rene Rodriguez, Carolin Seeling, Nada Al Shaaili, Shabnam Shaabani, Kristina Shiavone, Snehadri Sinha, Eleni M Tomazou, Marcel Trautmann, Maria Vela, Yvonne MH Versleijen‐Jonkers, Julia Visgauss, Marta Zalacain, Sebastian J Schober, Andrej Lissat, William R English, Nicola Baldini, Dominique Heymann

**Affiliations:** ^1^ Max‐Eder Research Group for Pediatric Sarcoma Biology Institute of Pathology Faculty of Medicine LMU Munich Munich Germany; ^2^ Division of Translational Pediatric Sarcoma Research German Cancer Research Center (DKFZ), Hopp Children's Cancer Center (KiTZ), German Cancer Consortium (DKTK) Heidelberg Germany; ^3^ Institute of Pathology Heidelberg University Hospital Heidelberg Germany; ^4^ Program in Solid Tumors and Biomarkers Foundation for the Applied Medical Research University of Navarra Pamplona Pamplona Spain; ^5^ Orthopedic Pathophysiology and Regenerative Medicine Unit IRCCS Istituto Ortopedico Rizzoli Bologna Italy; ^6^ Pediatric Soft Tissue Sarcoma Research Group German Cancer Research Center (DKFZ) Heidelberg Germany; ^7^ Department of Pediatrics and Children's Cancer Research Center (CCRC) Technische Universität München Munich Germany; ^8^ Children's Cancer Research Institute Vienna Austria; ^9^ Department of Pediatric Oncology/Hematology Charité‐Universitätsmedizin Berlin Berlin Germany; ^10^ Cellular Biotechnology Unit Instituto de Salud Carlos III Madrid Spain; ^11^ Centre for Craniofacial and Regenerative Biology King's College London London UK; ^12^ Department of Pediatrics University Hospital Erlangen Germany; ^13^ Division of Oncology Adhesion and Metastasis Laboratory Center for Applied Medical Research University of Navarra Pamplona Spain; ^14^ Institute of Biostructures and Bioimaging (IBB) Italian National Research Council (CNR) Turin Italy; ^15^ Department of Oncology and Metabolism University of Sheffield Sheffield UK; ^16^ Department of General, Visceral and Transplantation Surgery University of Heidelberg Heidelberg Germany; ^17^ Université de Nantes, Inserm, U1238 Nantes France; ^18^ Department of Pharmaceutical Technology Faculty of Pharmacy University of Coimbra Coimbra Portugal; ^19^ Instituto de Investigación Sanitaria del Principado de Asturias Oviedo Spain; ^20^ CIBER en oncología (CIBERONC) Madrid Spain; ^21^ Institute of Pathology Ulm University Ulm Germany; ^22^ Department of Drug Design University of Groningen Groningen The Netherlands; ^23^ Department of Oral and Maxillofacial Diseases University of Helsinki Helsinki Finland; ^24^ Division of Translational Pathology Gerhard‐Domagk‐Institute of Pathology Münster University Hospital Münster Germany; ^25^ Hospital La Paz Institute for Health Research (IdiPAZ) Madrid Spain; ^26^ Department of Medical Oncology Radboud University Medical Center Nijmegen The Netherlands; ^27^ Medical Center Duke University Durham NC USA; ^28^ University Children′s Hospital Zurich – Eleonoren Foundation Kanton Zürich Zürich Switzerland; ^29^ Department of Biomedical and Neuromotor Sciences University of Bologna Bologna Italy; ^30^ Université de Nantes Institut de Cancérologie de l'Ouest Tumor Heterogeneity and Precision Medicine Saint‐Herblain France

**Keywords:** bone sarcoma, molecular diagnostics, molecular medicine, soft tissue sarcoma, targeted therapy, Cancer, Molecular Biology of Disease, Musculoskeletal System

## Abstract

Sarcomas are heterogeneous and clinically challenging soft tissue and bone cancers. Although constituting only 1% of all human malignancies, sarcomas represent the second most common type of solid tumors in children and adolescents and comprise an important group of secondary malignancies. More than 100 histological subtypes have been characterized to date, and many more are being discovered due to molecular profiling. Owing to their mostly aggressive biological behavior, relative rarity, and occurrence at virtually every anatomical site, many sarcoma subtypes are in particular difficult‐to‐treat categories. Current multimodal treatment concepts combine surgery, polychemotherapy (with/without local hyperthermia), irradiation, immunotherapy, and/or targeted therapeutics. Recent scientific advancements have enabled a more precise molecular characterization of sarcoma subtypes and revealed novel therapeutic targets and prognostic/predictive biomarkers. This review aims at providing a comprehensive overview of the latest advances in the molecular biology of sarcomas and their effects on clinical oncology; it is meant for a broad readership ranging from novices to experts in the field of sarcoma.

## Graphical abstract

Sarcomas are heterogeneous and clinically challenging soft tissue and bone cancers. The current article comprehensively reviews recent advances in the molecular characterization of sarcoma subtypes, and describes novel therapeutic targets and biomarkers in this field.

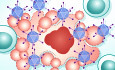

**Glossary**
Cancer stem cells (CSCs): Cells within the tumor found in very small fractions that are thought to be responsible for resistance to cancer treatments and thus relapse.
Cell dormancy: Stage in cancer progression during which tumor cells cease dividing but survive in a quiescent state while waiting for appropriate environmental conditions.
Chorioallantoic Membrane (CAM) models: Chick embryo CAM models used to study tumor formation, angiogenesis, and metastasis.
Circulating tumor cells (CTCs): Cells that leak into the vasculature or lymphatics from a primary tumor and are carried around the body in the blood circulation.
Epigenomic alterations: Heritable change that does not affect the DNA sequence but results in a change in gene expression.
Extracellular vesicles (EVs): Heterogeneous family of vesicles generated from different subcellular compartments and released into the extracellular space or the blood circulation.
Genomic alterations: Permanent modifications in the DNA sequence including somatic mutations, copy‐number variations (CNVs), and gene fusions.
Immunotherapy: Type of cancer treatment that aids the immune system to fight tumors.
Oncolytic viruses: Viruses that, by their intrinsic properties or through genetic engineering, specifically replicate in and kill cancer cells.
Orthotopic xenografts: Animal models based on the injection of tumor cell lines in the location where the tumors typically appear in humans.
Patient‐derived xenografts (PDXs): Animal model based on transplantation of human tumor biopsies that encompass tumor cells and the TME in immunodeficient animals.
Pediatric tumors: Tumors that typically arise between 0–14 years of age.
Precision medicine: Approach to patient care that allows physicians to select the treatments that are most likely to help patients based on a molecular understanding of their disease.
Sarcomas: Malignant neoplasms that originate from the skeleton or soft tissues.
Tumor microenvironment (TME): Cellular environment in which cancer cells reside encompassing the extracellular matrix and stromal cells (endothelial cells, fibroblasts, and immune cells)

## Epidemiology of sarcoma

**Figure 1:**
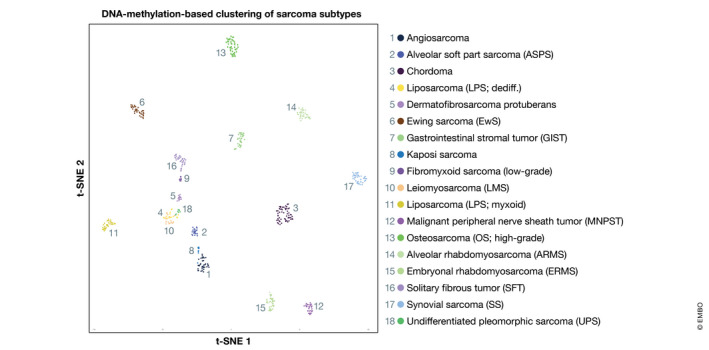
Diversity of sarcomas as highlighted by DNA methylation profiling t‐distributed stochastic neighbor embedding (t‐SNE) plot of *n* = 18 major sarcoma and soft tissue tumor subtypes based on genome‐wide DNA methylation profiling on Illumina EPIC arrays (Koelsche *et al*, 2018a,b). Web‐link to classifier: www.molecularsarcomapathology.org.

Although sarcomas are rare among adult malignancies, they represent 12–15% of all pediatric tumors (Stiller *et al*, 2013). Despite the implementation and continuous optimization of multimodal therapies, around one‐third of sarcoma patients still succumb to the disease. Historically, sarcomas have been clustered in two large subgroups, according to the anatomical site of occurrence—sarcomas of the skeleton and sarcomas of the soft tissues (hereafter referred to as “bone sarcomas” or “soft tissue sarcomas” [STSs], respectively). Both subgroups comprise a variety of histological subtypes, and recent technological advances have enabled to decipher a constantly increasing number of subtypes at the molecular level (Fig 1; Baldauf *et al*, 2018a; Koelsche *et al*, 2018a; Watson *et al*, 2018; Weidema *et al*, 2020). Table 1 summarizes the major sarcoma subtypes discussed in this review and their main features.

**Table 1:** Main sarcoma subtypes discussed in this review and their characteristics

Sarcoma subtype	Abbreviation	Main features
Bone sarcomas		
Chondrosarcoma^a^	CHS	• Localization: Cartilage, bone surface, or centrally in bone • Histopathology: Lobules composed of malignant chondrocytes entrapped in a chondroid matrix with calcified foci • Identified mutations of *IDH1/2*, *EXT1/2*
Ewing sarcoma^a^	EwS	• Localization: Long and flat bones (˜85%), extraskeletal sites (˜15%) • Histopathology: Undifferentiated small round cells; mostly strong membranous CD99 immunoreactivity and PAS‐positive cytoplasm • Harbor somatic *FET‐ETS* translocations (˜85% *EWSR1‐FLI1;* ˜10% *EWSR1‐ERG*; ˜5% rare subtypes)
Osteosarcoma^a^	OS	• Localization: Bone surface or centrally in bone • Histopathology: Neoplastic cells with mesenchymal morphology and frequent polymorphism (epithelioid, fusiform, round, spindled, etc.) associated with an extracellular osteoid matrix • Various subtypes including telangiectatic OS characterized by numerous hemorrhagic areas • Complex highly aneuploidy karyotypes with multiple chromosomal aberrations (numerical and structural) • Frequent *TP53* and *RB* mutations and numerous other mutations defining a “BRCAness” signature
Soft Tissue Sarcomas (STSs)		
Fibrosarcoma^a^		• Localization: Deep soft tissues of the extremities, trunk, and head & neck • Histopathology: Composed of monomorphic fibroblastic cancer cells in collagenous matrix
GastroIntestinal Stromal Tumors	GIST	• Localization: Gastrointestinal track (main site: stomach and small intestine) • Histopathology: broad morphological spectrum with mainly spindle cells and epithelioid cells (˜20% of cases) or mixed histology characterized by differentiation toward the interstitial cells of Cajal. Usually immunopositive for CD117 (KIT) and DOG1 • Harbor frequent activating mutations in *KIT* and *PDGFRA*
Leiomyosarcoma	LMS	• Localization: Most commonly detected in the peritoneum and uterus (rarely in bone) • Histopathology: Mesenchymal, spindle‐shaped cells with smooth muscle differentiation (SMA, desmin and h‐Caldesmon positivity) • Highly complex karyotypes with genomic instability
Liposarcoma^a^	LPS	• Localization: Variable (most commonly in the retroperitoneal space) • Histopathology: Cancer cells with variable adipocytic differentiation and heterogenous morphology embedded in a vascularized stroma (in case of myxoid LPS in myxoid stroma)
Rhabdomyosarcoma	RMS	• Localization: Variable • Histopathology: Mesenchymal phenotype with variable myogenic differentiation (usually positive for myogenin and MYOD)
Undifferentiated pleomorphic sarcoma^a^	UPS	• Localization: Most frequently in extremities • Histopathology: Undifferentiated cancer cells with a high degree of cellular atypia and pleomorphism
Synovial sarcoma	SS	• Localization: Mostly in deep soft tissues of the extremities • Histopathology: Spindle cells with variable mesenchymal and/or epithelial differentiation (i.e., monophasic/biphasic SS) • Harbor specific *SS18‐SSX1/2/4* fusion oncogenes

a The most common bone sarcoma and STS subtypes (WHO Classification of Tumours: Soft Tissue and Bone Tumours, 2020).

Among bone sarcomas, osteosarcoma (OS) is the most frequent subtype (Heymann, 2014). OS primarily affects adolescents and young adults, with the first and largest peak of incidence at age ~10–14 years. Coinciding with the pubertal growth spurt, the incidence rate of OS is 4 (3.5–4.6) for the range 0–14 years and 5 (4.6–5.6) for the range of 0–19 years per year per million persons (Ottaviani & Jaffe, 2009). The current standard of care was first introduced in the late 1970s and remains largely unaltered despite numerous efforts to improve outcomes (Rosen *et al*, 1976). Nowadays, patients with localized disease still face 5‐year overall survival rates < 70%, and < 20% of patients who develop metastatic disease or relapse survive > 3 years (Roberts *et al*, 2019). Ewing sarcoma (EwS) is included in the group of bone sarcomas because it is an aggressive sarcoma of both bone (~85% of cases) and soft tissue (~15% of cases), and because it has an incidence and survival rate similar to OS.

The STS subgroup comprises ~70–80% of all sarcomas with > 70 heterogenous histological subtypes (WHO Classification of Tumours: Soft Tissue and Bone Tumours, 2020). Although STSs represent < 1% of all cancers, they have the highest incidence among rare malignancies. Overall, the 5‐year survival for STS is estimated at ~57–62% and can vary widely depending on the disease stage and the complex interplay between anatomical site and STS subtype (Lyu *et al*, 2019). Unfortunately, the epidemiological data on specific STS subtypes are limited and frequently incomplete. National initiatives are ongoing to improve the databases, and they likely will benefit from the use of “big data” approaches. A recent review on the epidemiology of STSs in Italy and other European countries stated that they generally have an incidence of 6.27 and 4.71 cases per 100,000 inhabitants per year in Italy and Europe, respectively (Trama *et al*, 2019), with median ages at diagnosis of 58 and 63 years, respectively. Leiomyosarcoma (LMS), liposarcoma (LPS), and undifferentiated pleomorphic sarcoma (UPS; previously termed malignant fibrous histiocytoma) are the most common STS subtypes (WHO Classification of Tumours: Soft Tissue and Bone Tumours, 2020). A recent study in the Australian population reported that the incidence rate has almost doubled in the last 30 years (Bessen *et al*, 2019), which could be related to improved diagnostics or molecular pathology sub‐classification.

## The complex biology of sarcoma: How current knowledge may affect therapy

To date, targeted therapy of sarcomas has only been partially effective, possibly due to the existence of compensatory pathways, the intrinsically heterogeneous nature of sarcomas, and the complex interplay with the tumor microenvironment (TME; Brown *et al*, 2018). In the TME, multiple intermingled cell types coexist through complex heterotypic cellular interactions and communicate via a large array of paracrine signals. The heterogeneity of different cancer cell subpopulations is further modulated by the extracellular matrix, admixed with intra‐ and extracellular reactive elements, such as metabolites, oxygen tension, and pH.

### Impact of the tumor microenvironment on the stemness and behavior of sarcoma cells

**Figure 2:**
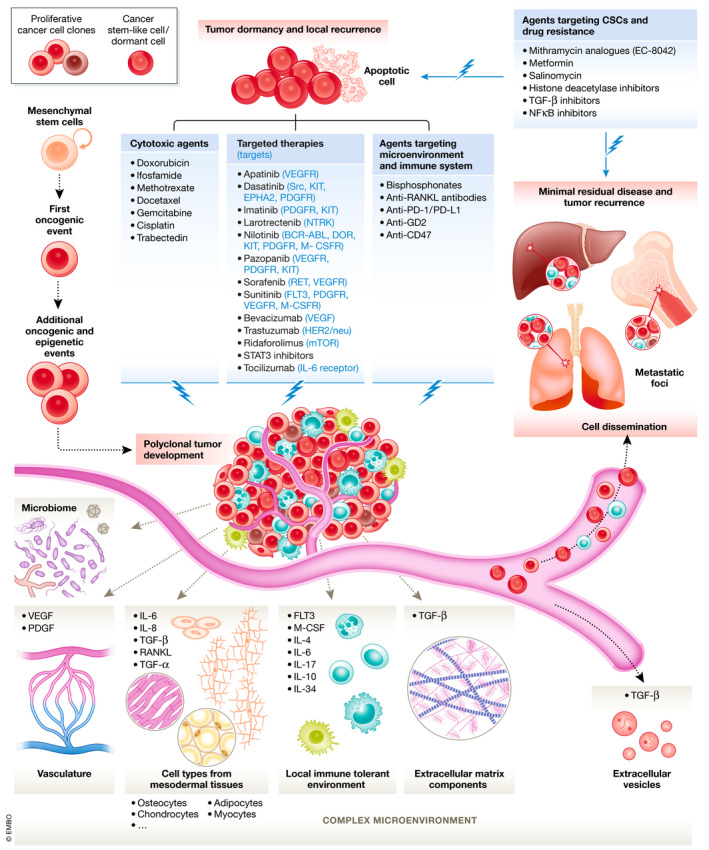
Biological features of sarcomas and therapeutic approaches Sarcoma development results from a complex biological process. Their natural history combines the emergence of a first oncogenic hit followed by secondary oncogenic and epigenetic events with a conjuncture of a permissive microenvironment composed by cell types from mesodermal tissues, immune infiltrate, vascular, and extracellular matrix components. Sarcoma cells interact with their close environment through direct contact, enhanced cytokine/growth factors/miRNA signaling under a soluble form or encapsulated in extracellular vesicles. Sarcoma cells are characterized by a phenotypic and genetic heterogeneity coming from the successive oncogenic/epigenetic events occurring during tumor development and by cancer cells acquiring stemness properties that become progressively quiescent. Sarcomas are prone to induce distant metastatic foci spread by circulating tumor cells and invading after extravasation appropriate metastatic niches. Cancer cells installed in distant organs can spread again and enrich other metastatic sites increasing the tumor heterogeneity and potentially drug resistances. Persisting cells after resection of the primary tumor or dormant cancer cells located in distant organs characterize the minimal residual disease and are responsible of tumor recurrences. A selection of approved and experimental treatments aimed to prevent tumor growth and/or dissemination is shown.

Similar to the “seed and soil” theory described for other malignancies, sarcoma cells evolve in a permissive milieu favoring their quiescence and drug resistance or their proliferation and aggressiveness. Sarcoma cells are embedded in a highly heterogeneous tissue context composed of immune cells, endothelial cells, pericytes, mesenchymal stem cells (MSCs), cancer‐associated fibroblasts (CAFs), and nerve fibers, all of which may influence their behavior and favor “stemness” properties. Cancer stem cells (CSCs) usually represent only a very small fraction of the tumor cell mass, yet their eradication is critical for improving drug response. Indeed, CSCs have a great potential for self‐renewal and develop protective mechanisms against conventional anti‐tumor treatments, thereby causing sarcoma relapse and metastasis (Abarrategi *et al*, 2016; Brown *et al*, 2017a; Fourneaux *et al*, 2019; Hatina *et al*, 2019). Common methods of isolating/enriching CSCs to model sarcoma heterogeneity *in vitro* include culturing floating three‐dimensional (3D)‐colonies (tumorspheres), cell sorting based on the expression of specific markers (i.e., CD133, ABCG2, CD44, CD184, STRO1, CD117, CD271, or aldehyde dehydrogenase 1), the ability to extrude fluorescent dyes (side populations), or the selective pressure induced by long‐term culturing with chemotherapeutic drugs. CSCs have been extensively characterized in both bone sarcomas and STSs (Salerno *et al*, 2013; Abarrategi *et al*, 2016; Brown *et al*, 2018; Genadry *et al*, 2018; Skoda & Veselska, 2018; Hatina *et al*, 2019; Schiavone *et al*, 2019; Fig 2).

Stemness in sarcoma is a fluctuating functional state orchestrated by the expression of pluripotency factors, such as OCT3/4, NANOG, KLF4, and, especially, SOX2 (Basu‐Roy *et al*, 2012; Maurizi *et al*, 2018; Skoda & Veselska, 2018; Sannino *et al*, 2019). The expression of these factors in sarcomas is oncogene‐driven and triggered by a combination of mutational and epigenetic events or by developmental programs (Rodriguez *et al*, 2012; Xiao *et al*, 2013). These events ultimately result in the deregulation of pathways that control stemness and differentiation, such as Hedgehog, Notch, Wnt/β‐Catenin, Hippo, or ALK (Graf Finckenstein *et al*, 2008; Naka *et al*, 2010; Riggi *et al*, 2010; Rodriguez *et al*, 2013; Basu‐Roy *et al*, 2015, 2016; Eid & Garcia, 2015; Tamaki *et al*, 2015; Abarrategi *et al*, 2016; Almazán‐Moga *et al*, 2017; Slemmons *et al*, 2017; Deel *et al*, 2018; Genadry *et al*, 2018; Hatina *et al*, 2019; Rodríguez‐Núñez *et al*, 2019; Schiavone *et al*, 2019; Trautmann *et al*, 2019). Alternatively, both stemness and aggressiveness can be regulated by the interaction with cells in the TME (Alfranca *et al*, 2015; Schiavone *et al*, 2019), or physical and chemical properties of the TME (i.e., hypoxia and extracellular acidosis) (Zeng *et al*, 2011; Alfranca *et al*, 2015; Avnet & Cortini, 2016; Avnet *et al*, 2017).

Several recent studies have focused on characterizing the sarcoma‐associated stroma and its effect on drug response (Tarnowski *et al*, 2010; Ehnman *et al*, 2013; Baglio *et al*, 2017; Cortini *et al*, 2017, 2019; Avnet *et al*, 2019). OS cells interact closely with MSCs, CAFs, osteoblasts, osteocytes, osteoclasts, chondrocytes, immune infiltrates, or components of the extracellular matrix to drive stemness‐promoting signaling (Avnet *et al*, 2008; Basu‐Roy *et al*, 2012; Zhang *et al*, 2013; Alfranca *et al*, 2015; Avnet & Cortini, 2016; Heymann *et al*, 2019). Moreover, MSCs/CAFs regulate tumor growth and metastasis through PDGFRα/β and MIF‐CXCR4/7 signaling, enhancing sarcoma aggressiveness via the secretion of inflammatory cytokines, exosomes (Miller *et al*, 2013; Cortini *et al*, 2016; Avnet *et al*, 2017; Baglio *et al*, 2017; preprint: Evdokimova *et al*, 2019), or metabolites that can fuel the oxidative metabolism of tumor cells (Bonuccelli *et al*, 2014). Metabolic fueling of sarcoma cells by stromal cells may be particularly relevant to sustain the energy demand of uncontrolled tumor growth and progression (Zhang *et al*, 2010; Ren *et al*, 2017; Gaude *et al*, 2018; Zhu *et al*, 2019). Consequently, the composition of the local TME has direct influence on the histological response to chemotherapy (Crenn *et al*, 2017). In addition, although axonogenesis has largely been neglected in sarcoma preclinical modeling so far, increasing evidence suggests that nerves in the TME may contribute to tumorigenesis, progression, and cancer‐associated pain in several sarcoma subtypes, such as fibrosarcoma, OS, EwS, LPS, and extraskeletal myxoid chondrosarcoma (CHS; Cain *et al*, 2001; Wacnik *et al*, 2005; Endo *et al*, 2008; Ghilardi *et al*, 2010; Kanojia *et al*, 2015; Moriarity *et al*, 2015; Shor *et al*, 2015; Brenca *et al*, 2019).

Moreover, the sarcoma TME may contain a specific microbiome (Nejman *et al*, 2020): A recent study described that bacterial DNA can be found in most CHSs. Bacteria were mostly intracellular and were detectable in immune and tumor cells (Nejman *et al*, 2020). Interestingly, metabolic functions related to intratumoral bacteria appeared tumor type‐specific; that is, degradation of hydroxyprolines by bacteria was enriched in CHSs (Nejman *et al*, 2020). Although more work is needed to decipher the precise role(s) of this symbiotic microenvironment, it is tempting to speculate that it could affect the stemness/differentiation and metabolic state of CHSs and possibly other sarcomas.

To date, several clinical and preclinical studies have reported treatments able to target the TME and/or CSCs in sarcomas (Abarrategi *et al*, 2016; Genadry *et al*, 2018; Schiavone *et al*, 2019) (Fig 2). The advent of techniques for single‐cell analysis, such as single‐cell DNA/RNA sequencing and spatial transcriptomics, will accelerate studying and modeling of sarcoma tissue heterogeneity and possibly lead to the identification of novel biomarkers and/or therapeutic targets.

### The immune infiltrate in sarcoma as a source of new therapeutic targets

**Figure 3:**
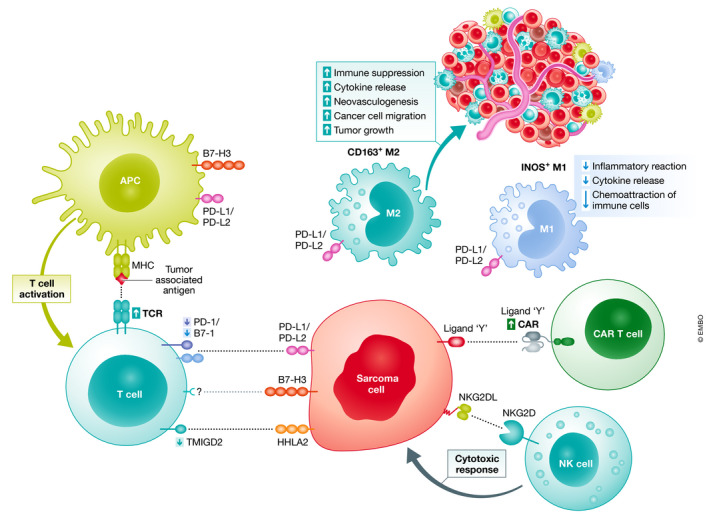
Sarcomas are characterized by an immune oasis Sarcomas are infiltrated by numerous immune cells, which are in some sarcoma subtypes deleterious by establishing an immune tolerant microenvironment that can be at the origin of innovative therapeutic approaches. In physiological condition, the adaptive immune system is activated by exogenous antigens leading to initiation of an effective immune response against the host at the origin of these antigens. Unfortunately, in most cases immune activation by tumor‐associated antigen is counterbalanced by inhibitory signals transmitted after the binding of immune checkpoint molecules (e.g., PD‐1) expressed by immune effectors to their ligands expressed by cancer cells such as PD‐L1. Macrophages also contribute to the immune surveillance in sarcomas with two main distinct subsets: M1 macrophages with pro‐tumor activities and M2 macrophages with anti‐tumor and immunosuppressive functions. This immune landscape has led to the development of immunotherapies including immune checkpoint inhibitors, activated NK cells, or genetically modified T lymphocytes (CAR T cells) in order to reactivate the tumor immune surveillance.

The TME of sarcoma cells is infiltrated by different immune cell populations (Fig 3). For example, OS tumor tissues are infiltrated by T lymphocytes (tumor‐infiltrating lymphocytes, TILs) in a very high percentage of patients, mainly expressing CD8^+^ (Théoleyre *et al*, 2005; Palmerini *et al*, 2017), and both TILs and tumor cells showed a high expression of HLA‐DR compared with other, non‐malignant bone tumors (Trieb *et al*, 1998). In preclinical models, CD8^+^ TILs are cytotoxic against allogeneic tumor cells (Théoleyre *et al*, 2005), and the number of CD8^+^ or CD8^+^/TIA1^+^ TILs correlates positively with longer survival in patients (van Erp *et al*, 2017; Gomez‐Brouchet *et al*, 2017; Palmerini *et al*, 2017). Similarly, in a small percentage of tumors, FOXP3^+^ (regulatory T cells, Tregs), and Arginase^+^ (myeloid‐derived suppressor cells, MDSCs), immune‐suppressive infiltrating cells were detected (Fritzsching *et al*, 2015; Palmerini *et al*, 2017). Notably, the CD8^+^/FOXP3^+^ ratio had a positive prognostic value (Fritzsching *et al*, 2015). Furthermore, a high pretreatment ratio of infiltrating neutrophils to lymphocytes, high levels of C‐reactive protein, Glasgow prognostic score, platelet–lymphocyte ratio score, and lymphocyte‐monocyte ratio or systemic absolute leukocyte counts in post‐therapeutic early recovery are independent prognostic markers (Moore *et al*, 2010; Liu *et al*, 2016; Vasquez *et al*, 2017).

Sarcomas are also frequently infiltrated by macrophages, which represent the main immune infiltrate and a highly heterogeneous population (Toulmonde *et al*, 2018; Mu *et al*, 2019; Stahl *et al*, 2019). Macrophage subpopulations are composed of a balance between immune‐stimulatory M1 and immune‐suppressive M2 macrophages that can be dysregulated in sarcomas. Both subpopulations are CD68^+^ and can be distinguished by the INOS and CD163 expression in M1 and M2 macrophages, respectively (Jayasingam *et al*, 2020). However, their roles are complex, as revealed by the functional discrepancy observed according to the given sarcoma subtype. Indeed, CD163^+^ is required for their protumoral activities (Shiraishi *et al*, 2018) and is a prognostic marker for specific sarcoma subtypes such as embryonal rhabdomyosarcoma (ERMS; Kather *et al*, 2019), whereas in OSs, CD163^+^ M2 macrophages are proangiogenic, facilitating cancer cell extravasation and promoting the metastatic process (Dumars *et al*, 2016; Han *et al*, 2016; Gomez‐Brouchet *et al*, 2017). Conflicting results showed a positive association of tumor‐associated dendritic cells (CD1a^+^) and macrophages with either a worse disease‐free survival (Koirala *et al*, 2016) or inhibition of metastases (Buddingh *et al*, 2011). However, their phenotype has not been fully characterized.

Sarcomas driven by reciprocal fusion oncoproteins, such as EwS, generally exhibit a low immune infiltrate, constituting so‐called “cold” tumors. Few available studies have demonstrated that TILs and dendritic cells are quite rare (immune desert) and that programmed death‐ligand 1 (PD‐L1) expression is usually low (Spurny *et al*, 2018). The presence of infiltrating macrophages has been associated with poorer overall survival (Vakkila *et al*, 2006), and elevated levels of circulating proinflammatory factors (e.g., interleukin 6, IL‐6) correlate with tumor‐associated fever at advanced stages (Lissat *et al*, 2015), implying the recruitment of immunosuppressing myeloid dendritic cells, macrophages, and other inflammatory cells at the tumor site (preprint: Evdokimova *et al*, 2019).

For STSs, only a very few recent reports have aimed to determine the “hot” or “cold” tumor immunophenotypes and their potential as biomarkers for response to therapy (Galon & Bruni, 2019). Kim *et al* reported the presence of PD‐1^+^ and PD‐L1^+^ TILs at rates of 65% and 58%, respectively, in various STS subtypes (Kim *et al*, 2013a). Similarly, the infiltrations of PD‐L1‐expressing macrophages and lymphocytes were observed in 58% and 30%, respectively, of 50 analyzed STS samples (D'Angelo *et al*, 2015), and the PD‐L1 expression was associated with a higher density of CD3^+^ PD‐1^+^ TILs, a higher tumor grading, and a lower overall survival (Orth *et al*, 2020). PD‐L1 was also expressed by tumor cells in 12% of cases, with the highest prevalence in gastroIntestinal stromal tumors (GISTs). Finally, the detection of low CD3^+^ or CD4^+^ TILs was significantly correlated with better overall survival by a univariate analysis (D'Angelo *et al*, 2015). However, recent reports have provided a more panoramic view of PD‐1 and PD‐L1 expression in larger series of STS and revealed that most STS subtypes show expression of both factors (Dancsok *et al*, 2019; Orth *et al*, 2020). However, the bioclinical relevance of PD‐1 and PD‐L1 (e.g., prognostic value) remains controversial in sarcomas, mainly due to their high heterogeneity (Fujii *et al*, 2014; Nduom *et al*, 2016; Nowicki *et al*, 2017).

Collectively, the immune infiltrates observed in sarcomas offer a rich opportunity for implementation of immunotherapeutic approaches in sarcomas. Yet, a complete and more standardized immune score may help to better understand the different immunophenotypes related to each sarcoma subtype and to improve immunotherapeutic approaches.

### Models for studying the biology of sarcomas

Human cancer cell lines have become the cornerstone of cancer research. However, the accumulation of (epi‐)genetic mutations over time and across laboratories can have crucial implications when investigating new treatments as shown in carcinoma cell lines (Liu *et al*, 2019), since they affect drug response (Ben‐David *et al*, 2018). Whether this holds true for translocation‐driven sarcomas, such as alveolar rhabdomyosarcoma (ARMS), EwS, myxoid LPS, and SS, which display rather “silent” genomes, remains to be determined. Yet, the use of low‐passaged primary cell lines can prevent accumulation of mutations: A recent study of CHS patient samples and their derived cell lines characterized the genetic drift process of primary cell lines after 20–34 *in vitro* cell culture passages (Rey *et al*, 2019). Although the adaptation of tumor cells to *in vitro* cell culture is accompanied by additional genetic mutations, these rather low‐passaged CHS cell lines retained the most relevant mutations of the patient's founder clone (Rey *et al*, 2019).

For preclinical modeling of sarcoma, 3D culture has recently emerged as a tool for better prediction of drug efficacy and development of precision medicine approaches (Vaira *et al*, 2010; Santoro *et al*, 2015; Bregenzer *et al*, 2019). These 3D models include microfluidic devices, bioprinted cell‐enriched structures with tailorable biomechanical properties, and well‐defined tumoroids (Murphy & Atala, 2014), which contain different cell types, defined gradients of bioactive factors, and “physiological” biomaterials to precisely recapitulate the natural TME (Ma *et al*, 2018). This will help to elucidate the mechanical cross‐talk between sarcoma cells and “normal” cells (including vasculature and immune cells) (Huang *et al*, 2014; Datta *et al*, 2017), as well as components of the extracellular matrix (Doraiswamy *et al*, 2007; Pavlou *et al*, 2019). However, although a recent study has successfully employed a mineralized 3D bone model to evaluate the effect of the small‐molecule elesclomol on EwS cells (Marchetto *et al*, 2020), 3D models for the study of sarcoma are still in their infancy (Barron *et al*, 2004, 2005).

*In vivo*, the chick chorioallantoic membrane (CAM) assay is a valuable option due to its low costs and relatively easy implementation. CAM assays have been employed to study sarcoma angiogenesis, fibroblast infiltration, tumorigenesis, tumor invasion, and metastasis in CHS, EwS, fibrosarcoma, LPS, and OS (Sys *et al*, 2013; Patil *et al*, 2014; Manjunathan & Ragunathan, 2015; Cimpean *et al*, 2018; Kunz *et al*, 2019; Perut *et al*, 2019; Steinestel *et al*, 2020). Numerous additional *in vivo* models of inducible or spontaneous sarcomas have been described in non‐mammalian vertebrates (e.g., zebrafish; Leacock *et al*, 2012; Mohseny *et al*, 2012; Brown *et al*, 2017b; Hayes & Langenau, 2017; Ignatius *et al*, 2018; Fleming *et al*, 2019) and in mammalians (e.g., mouse, rat, and dog; Cannon, 2015; Jacques *et al*, 2018; Castillo‐Tandazo *et al*, 2019; Pomella & Rota, 2020). Genetically modified zebrafish and xenotransplantation of human sarcoma cells in fish were simultaneously proposed. Their main advantages are (i) their small size, allowing the maintenance of many animals at low costs; (ii) their high rate of proliferation (> 200 embryos per pairing); (iii) *ex utero* development of embryos, facilitating cell transplantations; (iv) their transparency, which facilitates non‐invasive and repeated imaging; (v) the possibility of imaging at the single‐cell level; (vi) studies of human cells and host factors facilitated by the use of transgenic lines; (vii) no immune rejection in early cell transplantation; and (viii) facilitation of high‐throughput drug screening due the animals’ permeability to small molecules through diffusion. Yet, the lack of specific organs (e.g., lungs) and the difference with human TME are two major limitations of zebrafish models (Mohseny *et al*, 2012; Brown *et al*, 2017b; Hayes & Langenau, 2017).

Genetically engineered mouse models (GEMMs) are considered reliable models for studying cancer development. Indeed, by inducing the formation of spontaneous tumors mimicking the natural history of human pathologies, GEMMs are privileged models to functionally identify and characterize molecular drivers or genetic initiator events of the disease (Kersten *et al*, 2017). While EwS, for which no *bona fide* GEMMs have been developed to date, is an exception among sarcomas, numerous GEMMs of bone sarcomas (for reviews, see ref. Jacques *et al*, 2018, 2019) and STSs (for review, see ref. Dodd *et al*, 2015) have been described. The first GEMM overexpressed the AP‐1 transcription factor c‐Fos in murine osteoblasts, which led to the development of OS without inducing metastatic foci (Grigoriadis *et al*, 1993). More recent models include deletion of *Tp53*, *Rb*, *Prx‐1*, or *Prkar1a;* overexpression of Sonic Hedgehog signaling components; or targeting *Apc* and *Twist*, and lead to the formation of metastatic OS (Jacques *et al*, 2018). Similarly, conditional loss of *Tp53* or *Ink4a/Arf* in an *Ext1*‐driven GEMM results in the formation of CHS (de Andrea *et al*, 2015).

GEMMs of STSs were also developed (Dodd *et al*, 2015). For example, the conditional *Pax3‐Fkhr* knock‐in allele is associated with the development of ARMS with a frequency that can be increased by the loss of function of *Ink4a/ARF* and *Tp53* (Keller *et al*, 2004). In addition, ERMS can be induced from the adipocyte lineage by adipocyte‐restricted activation of Hedgehog signaling through constitutive expression of an active *Smoothened* allele (Hatley *et al*, 2012). The latter model has also helped to demonstrate that Hedgehog signaling drives aberrant expression of myogenic specification factors, which may induce ERMS from non‐myogenic endothelial progenitors (Drummond *et al*, 2018). More recently, GEMMs for sarcomas have been obtained by CRISPR‐*Cas9* technology (Huang *et al*, 2017).

Xenografts are alternatives to GEMMs and can be obtained by injection of tumor cells into immunodeficient mice. Xenografts are relatively easy to generate and highly reproducible (Picarda *et al*, 2010; Gambera *et al*, 2018; Jacques *et al*, 2018), but cannot fully recapitulate the TME of many sarcoma subtypes, and only rarely give rise to spontaneous metastases (Jacques *et al*, 2018). In this context, orthotopic xenografts obtained through injection of a suspension of tumor cells into the para‐ or intraosseous site for OS and EwS modeling (Hauer *et al*, 2013; Lamora *et al*, 2014; Stewart *et al*, 2014; Ségaliny *et al*, 2015; Baglio *et al*, 2017), or through intramuscular injection for the modeling of “soft tissue EwS” (Jaboin *et al*, 2002; Merchant *et al*, 2004), more closely recapitulated the TME of the respective tumor histotype. Similarly, early passage patient‐derived xenografts (PDXs) constitute a powerful tool for preserving the TME, histology, and genetic profiles of sarcomas (Hoffman, 2015; Stewart *et al*, 2017). PDXs are obtained through subcutaneous or orthotopic implantation of small fragments of tumors isolated from patients in immunodeficient mice. However, so far, only few studies have been published on PDXs in sarcoma due to the low success rate of the engraftment, the complex implantation procedure (Stewart *et al*, 2017; Nanni *et al*, 2019; Rainusso *et al*, 2019), and the costs required for the stabilization of the model, which may require up to a year (Nanni *et al*, 2019).

## Current standard therapies for sarcomas

The therapeutic care of bone sarcoma and STS patients requires specialized sarcoma units. In fact, treatment in such specialized centers has been shown to result in improved surgical and oncologic outcomes (Blay *et al*, 2017). In addition, due to the potentially devastating consequences that can arise from poorly performed biopsies, biopsies of lesions suspected of being a sarcoma should be carried out in (or directed by) a specialized center (Mankin *et al*, 1982; Potter *et al*, 2008; Pretell‐Mazzini *et al*, 2015; Traub *et al*, 2018). The cornerstone of bone sarcoma and STS management is surgical resection of the primary tumor, which is typically accompanied by neoadjuvant and/or adjuvant chemotherapy and/or irradiation. Radiation therapy contributes to local control of tumor growth with positive margins or high‐grade STS (Kim *et al*, 2008). Chemotherapy regimens of bone sarcomas (e.g., OS, EwS) combine doxorubicin, cisplatin, methotrexate, and ifosfamide administered before and/after surgery for 6–12 months (Brown *et al*, 2018). Similarly, systemic treatments of STSs are mainly based on anthracyclines (e.g., doxorubicin) alone or in combination with an alkylating agent (e.g., ifosfamide) (Judson *et al*, 2014; Gómez & Tsagozis, 2020; Smrke *et al*, 2020). Interestingly, the use of adjuvant chemotherapy or radiotherapy may be defined by biological risk factors in high‐risk STSs (Sundby Hall *et al*, 2018). Although systemic therapy is the treatment of choice in metastatic disease (Meyers, 2015), resection of the primary tumor may still be performed with palliative intent, or rarely, in combination with resection of oligometastatic disease (Blakely *et al*, 2015). Wide margin surgery then remains the crucial technical approach in sarcoma treatment (Patrikidou *et al*, 2011).

For bone sarcomas, studies have demonstrated that oncologic outcomes of OS and EwS are similar between limb salvage and amputation when wide margins are achieved (Simon *et al*, 1986; Rougraff *et al*, 1994; Alamanda *et al*, 2012; Jauregui *et al*, 2018). Thus, the current standard of care is limb salvage surgery if preservation of neurovascular structures allows reconstruction of a functional extremity (Yang *et al*, 2017). Special considerations are made for limb reconstruction in the growing child, such as the use of growing prostheses, vascularized autografts, or van Nes rotationplasty. The choice of (neo)adjuvant treatment modalities is largely driven by the histological subtype: For instance, OS and EwS are usually chemosensitive and treated with neoadjuvant and adjuvant chemotherapy to decrease the risk of systemic disease progression, while STSs are frequently treated with neoadjuvant radiation therapy to decrease the risk of local recurrence (Gaspar *et al*, 2015; Brown *et al*, 2018; Le Cesne, 2018; Ray‐Coquard *et al*, 2018; Fig 2). In contrast, high‐grade CHS is largely resistant to existing chemo‐ and radiotherapies; thus, achieving a wide margin resection is currently the best option for prevention of disease progression (Reed *et al*, 2017; Brown *et al*, 2018; Whelan & Davis, 2018).

GIST is one of the STS subtypes for which the therapeutic development has been the most spectacular (Farag *et al*, 2020). For instance, up to 85% of patients with advanced GIST benefit from imatinib treatment (Blay, 2011). In fact, 90% of GISTs harbor driver mutations in the KIT proto‐oncogene receptor tyrosine kinase (*KIT*) and platelet‐derived growth factor receptor alpha (*PDGFRA*), which can be targeted by tyrosine kinase inhibitors (TKIs). Their therapeutic efficacy is directly linked to the type of mutation, and consequently, the acquisition of secondary mutations can result in drug resistance (see section “Resistance to targeted therapies”), which remains the most significant challenge in the treatment of locally advanced and metastatic GIST (Li & Raut, 2019). However, even fourth‐line therapy with TKIs may still be effective in advanced GIST (Blay *et al*, 2020).

Yet, the mostly moderate efficacy of any second‐line treatment for the majority of relapsed bone sarcomas and STSs highlights the need for intensified research to identify novel targets and improved preclinical models to predict drug response in molecularly defined cohorts of patients suffering from refractory and/or recurrent disease.

## Mechanisms of drug resistance

Chemoresistance has been largely associated with the expression of specific detoxifying molecules, such as efflux pumps (ATP‐binding cassette (ABC) family proteins or ALDH enzymes), as it has also been recently demonstrated for CSCs (Lohberger *et al*, 2012). In particular, P‐glycoprotein is a 170 kDa transmembrane energy‐dependent efflux pump encoded by the *MDR1* gene. Its expression leads to a multidrug resistance phenotype rather than an increased biological aggressiveness (Scotlandi *et al*, 1996; Baldini, 1997), which is associated with decreased event‐free survival in OS patients (Baldini *et al*, 1995) and in a small percentage of STS patients (Serra *et al*, 1996), and has also recently been found in bone sarcoma PDXs (Nanni *et al*, 2019).

Besides P‐glycoprotein, additional drug resistance mechanisms are caused by tumor heterogeneity arising from high DNA repair capacity, deregulation of apoptotic factors, adoption of a quiescent state (Honoki *et al*, 2010; Abarrategi *et al*, 2016; Martinez‐Cruzado *et al*, 2016; Roundhill *et al*, 2019; Vallette *et al*, 2019), drug delivery failure, the epithelial–mesenchymal transition (EMT) (Sannino *et al*, 2017), increased autophagy (Xiao *et al*, 2018), enrichment of CSCs (Eyler & Rich, 2008), protective signaling traits after chemotherapeutic treatment (Martins‐Neves *et al*, 2016; Yu *et al*, 2016), and immune evasion (Vasan *et al*, 2019).

In addition, resistance to conventional TKIs (e.g., imatinib) is associated with secondary mutations of *KIT* or *PDGFRA* in GIST (see section “Resistance to targeted therapies”). To overcome such acquired resistance, “switch pocket inhibitors” have been developed (Blay *et al*, 2020). A switch pocket inhibitor has the same target as the conventional inhibitors but acts like a light switch that deactivates cell signaling associated with the targeted receptor via blocking conformational activation of the kinase. For example, ripretinib targets KIT, PDGFRα/β, kinase insert domain receptor (KDR), and colony‐stimulating factor 1 receptor (CSF1R alias C‐FMS) and has been developed to overcome the TKI resistance occurring in GIST patients. The Asp842Val (D842V) mutation of *PDGFRA* was identified as the primary driver mutation in 5–6% of GISTs, which are refractory to all currently approved TKIs (Corless *et al*, 2005). The D842V mutation is located in the exon 18 encoding the *PDGFRA* activation loop and modifies the protein conformation to a “constitutive” active form.

Avapritinib is a new TKI designed on the base of its selectively property to target the active conformation of *KIT* and *PDGFRA*. A phase I clinical trial (ClinicalTrials.gov No. NCT02508532) has recently assessed its safety, tolerability, and anti‐tumor activity (Heinrich *et al*, 2020). Interestingly, 9% of complete response and 79% a partial response was observed. Ripretinib—an inhibitor of all known *KIT* and *PDGFRA* mutations—forces the switching of the mutated receptors to assume the “off” position. A recent double‐blind, randomized, placebo‐controlled, phase 3 clinical trial (ClinicalTrials.gov No. NCT03353753) showed that ripretinib significantly improved the progression‐free survival with an acceptable safety profile in patients suffering from advanced GIST resistant to approved treatment (Blay *et al*, 2020).

Similarly, the classification of *BRAF* mutations, the knowledge about dysregulated signaling pathways and dysregulated circuitries related to these mutations, and the function of BRAF in sarcoma led to the development of new therapeutic options to overcome resistance to conventional chemotherapy. For instance, the BRAF V600E mutation was recently identified as a potential therapeutic target in a small subset of SS (Watanabe *et al*, 2020). It is interesting to note that resistance to *BRAF* mutation inhibitors may be overcome by combining BRAF inhibitors with EGFR, PI3K, mTOR, MEK, RTK, HGF, and MET inhibitors, leading to the targeting of the MAPK and PI3K‐AKT‐mTOR signaling pathways (Liu *et al*, 2020). CX‐6258 is a pan‐Pim kinase inhibitor selected for its potent activity against sensitive and resistant cancer cells to RAF/MEK inhibitor (Haddach *et al*, 2011).

Using a KINOMEscan assay platform, haspin kinase was identified as a target of CX‐6258. The inhibition of haspin reduced cancer cell proliferation and regulated the immune system by increasing the frequency of interferon γ (IFNγ)‐producing CD8^+^ T cells and reducing the number of Tregs *in vivo* (Melms *et al*, 2019). Interestingly, the haspin kinase inhibitor can overcome RAF/MEK inhibitor‐resistant cancer cells and shows anti‐tumor effects in EwS (Melms *et al*, 2019). Acquired resistance to cisplatin observed in OS patients is associated with a poor prognosis (Higuchi *et al*, 2019). Peroxisome proliferator‐activated receptor gamma (PPARγ) was reported to enhance the efficacy and overcome resistance to cisplatin in various oncological entities and exhibits similar properties in OS (Higuchi *et al*, 2019).

The cell differentiation state also affects drug sensitivity (Dawson *et al*, 2020). A subpopulation of RMS cells that expressed *MYOD1* and *NOG* exhibited primary resistance to vincristine and doxorubicin, which can be partly overcome by the combination of 12‐O‐tetradecanoylphorbol‐13‐acetate (TPA) and an enhancer of zeste homolog 2 (EZH2) inhibitor (GSK126) (Dawson *et al*, 2020). EZH2 is an epigenetic drug acting as a histone methyltransferase inhibitor that has been recently approved for metastatic or locally advanced epithelioid sarcoma (Rugo *et al*, 2020). The elimination and recycling of damaged proteins and organelles are driven by autophagy, which provides energy to the cells. Autophagy can be activated by chemotherapy and can promote increased chemosensitivity, as well as drug resistance in OS (Camuzard *et al*, 2019; Liao *et al*, 2019). Thus, drugs regulating autophagy may be an option to overcome drug resistance in the future.

### Cell dormancy and recurrence

The risk of recurrence in oncology is associated with the persistence of cancer cells, which are not clinically/biologically detectable after resection of the primary tumor (Arlt *et al*, 2013). The latency without any detectable disease varies according to the clinical condition (e.g., histological grade and subtype) and depends on cancer cells characterized by slow cycling, low metabolism and fitness, and consequently, long‐term survival mechanisms (Vallette *et al*, 2019). Awakened cancer cells re‐acquire an active state, with capacities of proliferation and spreading to distant sites, and they define the minimal residual disease (Riethmüller & Klein, 2001). Dormant cells have been identified in several sarcoma subtypes, including fibrosarcoma (Dobson & Dickey, 1956; Varani *et al*, 1981; Cao *et al*, 1998), LPS (Almog *et al*, 2006; Rogers *et al*, 2014), RMS (Kimura *et al*, 2002), Kaposi sarcoma (Indraccolo *et al*, 2006), and OS (Naumov *et al*, 2006; Shimizu *et al*, 2014; Avril *et al*, 2016a,b; Guo *et al*, 2017). These rare dormant cells exhibit stemness properties (Visvader, 2011), and they have been related to drug resistance (De Angelis *et al*, 2019; Smith & Macleod, 2019; Vallette *et al*, 2019). The emergence of dormant cells is a conserved biological process linked to cell survival and controlled by multiple parameters, including genetic and epigenetic alterations, clonal cell evolution, cell–matrix interactions within the TME (e.g., immune tolerance), and diversity/heterogeneity. No specific molecular signature of dormant sarcoma cells has yet been identified. The most recent molecular approaches (e.g., single‐cell RNA sequencing, RNA/DNA methylation profiling) should lead to the identification of their specific molecular profile and of the molecular drivers of this state. For instance, myeloma dormant cells are switched “on” by engagement with osteoblastic cells and switched “off” by active osteoclasts (Lawson *et al*, 2015), which illustrates the clinical interest of targeting cell dormancy also in the context of bone sarcomas and STSs (Endo & Inoue, 2019; Recasens & Munoz, 2019; Tellez‐Gabriel *et al*, 2019).

### Resistance to targeted therapies

TKIs are the largest class of targeted therapies approved by the Food and Drug Administration (FDA). In particular, GIST commonly shows activating mutations in the receptor tyrosine kinases *KIT* and *PDGFRA*. While physiological KIT or PDGFRα signaling are involved in cell differentiation and survival, activating mutations in both genes results in constitutive ligand‐independent receptor activation, leading to GIST tumorigenesis. TKIs are the standard of care in the primary treatment of GIST, and imatinib is the most commonly used compound (Casali *et al*, 2018). The resistance toward TKIs in GIST is mainly related to secondary mutations of *KIT* (Li & Raut, 2019; Napolitano & Vincenzi, 2019), but can also be triggered by *PDGFRA* mutations (Lim *et al*, 2008; Kalfusova *et al*, 2019).

In non‐GIST STSs, the currently approved targeted therapies are limited to the multi‐target TKI pazopanib, which targets VEGFR‐1, VEGFR‐2, and VEGFR‐3, PDGFRα and PDGFR‐β; and KIT (Lee *et al*, 2019). It has been demonstrated that anti‐angiogenic TKIs, including pazopanib, do not succeed in targeting sarcoma stem cells (Canter *et al*, 2014), whereas treatment with pazopanib in a human SS model promotes the development of resistance (Lanzi *et al*, 2019). Despite a strong inhibition of the main target of pazopanib, PDGFRα/β, the activation of the AKT and ERK signaling pathways was only partially impaired, possibly due to the over activation of other tyrosine kinase receptors, including the insulin‐like growth factor receptor type 1 (IGF1R) and insulin receptor (IR). Similarly, in another SS cell line, the presence of an *NRAS* mutation sustained ERK activation and caused resistance to pazopanib treatment (Lanzi *et al*, 2019). Thus, a combination treatment with either an IGF1R/IR inhibitor or a MEK inhibitor has been suggested to restore the inhibition of the PDGFRα/β pathways and effectively promote apoptosis (Lanzi *et al*, 2019). Phosphoproteomic profiling of pazopanib‐resistant cells identified the inhibition of HSP90 as a therapeutic route to overcome resistance (Vyse *et al*, 2018).

These findings highlight the importance of patient‐specific tumor profiling to identify the underlying activated signaling pathways, thereby avoiding the “one‐size‐fits‐all” paradigm and moving toward personalized, multi‐line, and patient‐specific treatment regimens (Wilding *et al*, 2019). Biomarker‐guided basket trials, such as the CREATE trial, which evaluates multiple disease types with a common oncogenic driver matched to a specific targeted therapy, may be considered in this respect (Péron *et al*, 2019). Moreover, characterization of interpatient pharmacokinetic variability will be a valuable tool to predict and overcome the development of resistance (Cardoso *et al*, 2020).

### Other types of resistances

Several other indirect mechanisms of drug resistance in sarcoma have been identified, such as the formation of abnormal TME, hypoxia, and acidosis. Elevated levels of hypoxia and hypoxia‐inducible factor 1α (HIF1α) in human sarcomas correlate with tumor progression and radiation resistance (Kim *et al*, 2013b). In particular, in STS, HIF1α expression was found in 25.5% of tumors and was associated with both shorter overall survival and progression‐free survival (Kim *et al*, 2015). Moreover, translational activation of HIF1α by YB‐1 was found to promote metastasis in preclinical models of EwS, OS, and RMS (El‐Naggar *et al*, 2015). Similarly, in OS, hypoxia was responsible for the induction of the Wnt/β‐catenin signaling pathway and resulted in 6–13 times more cell resistance to doxorubicin‐mediated toxicity than under normoxic conditions (Roncuzzi *et al*, 2014; Scholten *et al*, 2014). In EwS, hypoxia has been found to protect tumor cells against anticancer drugs, while suppression of HIF1α enhanced drug‐induced apoptosis (Kilic *et al*, 2007). Accordingly, metabolic characterization, including hypoxic phenotypes, may help to identify specific treatment modalities in OS, other bone sarcomas, and STSs (Eary *et al*, 2011; Campanile *et al*, 2013). Along these lines, a recent pilot study characterized different metabolic parameters in a small group of STS patients using specific positron emission tomography (PET) agents to assess the individual risk associated with biological characteristics of the tumors (Wolsztynski *et al*, 2018).

Tumor acidosis is a metabolic adaptation observed in cancers and characterized by the fermentation of glucose to lactic acid. This process occurs in the presence of oxygen and is called aerobic glycolysis or Warburg effect. This adaptative mechanism modulates the drug sensitivity and leads to drug resistances by intrinsic (e.g., modulation of the mutational profile driven by a cell adaptation to stress) or extrinsic (e.g., structural/functional modulation of drugs induced by the local pH modifications) mechanisms (Kolosenko *et al*, 2017). Indeed, the pH of the local microenvironment regulates the passive diffusion of small molecules such as cancer drugs across biological barriers by modulating charged components of cell membranes, process named ion trapping or pH‐partitioning (Scott *et al*, 2017). Many cancer drugs are ionizable molecules containing weak bases or acids in their structure and are subjected to pH‐partitioning resistance (Zhitomirsky & Assaraf, 2016). That is the case for doxorubicin (weak base compound) in OS, which is trapped in the acidic extracellular microenvironment and consequently cannot target cancer cells (Avnet *et al*, 2016). On the contrary, the cytotoxic effects of cisplatin (weakly acidic drug) are increased in OS by the local tumor acidosis, which favors its neutral form and then facilitates its passive diffusion across the cell membranes (Avnet *et al*, 2016). In the cytoplasm**,** cisplatin is ionized by the low alkaline pH and trapped in the cell. A similar phenomenon was described in RMS, and the diffusion of weak base drugs across cell membranes and their sequestration in the lysosomal compartment are facilitated by ion trapping (Salerno *et al*, 2014; Zhitomirsky & Assaraf, 2016).

## Molecular signatures of sarcomas: Effects on diagnosis and prognosis

In past decades, an unbiased and systematic search for gene fusions combined with unsupervised gene expression and (epi)genetic analyses of different sarcoma subtypes led to better classification systems (WHO Classification of Tumours: Soft Tissue and Bone Tumours, 2020). In addition, these molecular signatures provide information about the biology of these tumors, reflecting both the characteristics of the sarcoma's cell of origin and the activated pathways driving the malignant phenotype (Taylor *et al*, 2011).

### Genomic and transcriptomic alterations

The Cancer Genome Atlas (TCGA) Research Network reported a recent analysis of 206 adult STSs representing six major subtypes (Cancer Genome Atlas Research Network, 2017). Here, the authors showed that common sarcomas (except for SS) are characterized by a high number of copy‐number variations (CNVs) and recurrent point mutations in relatively few genes, such as *TP53*, *ATRX,* and *RB1*. Importantly, specific genomic and transcriptomic alterations also define molecular subtypes, which are associated with patient outcome (Cancer Genome Atlas Research Network, 2017). Other studies have identified whole‐genome duplication as a cause of the structural complexity of UPS (Steele *et al*, 2019), and *CDKN2A* alterations as a predictor of worse overall survival across sarcoma subtypes (Bui *et al*, 2019). Integrated analysis of genomic and transcriptomic data confirmed the mutational profiles of STSs and identified PDGFRα as a putative target in complex karyotype STSs (Kim *et al*, 2018). Indeed, a PDGFRα*‐*blocking antibody (olaratumab) in combination with doxorubicin showed promising results for non‐GIST STS treatment (Klug & Heinrich, 2017). Given the widespread presence of *CDK4*‐amplification/high expression and *CDKN2A* loss across sarcomas subtypes, CDK4 inhibitors such as palbociclib are also a promising strategy in *RB‐*positive tumors (Dickson *et al*, 2013). It is noteworthy that ATRX has been shown to be required for response to CDK4 inhibitors in LPS, providing a potential biomarker for upcoming clinical trials (Kovatcheva *et al*, 2015; Cancer Genome Atlas Research Network, 2017). Integration of genomic and transcriptome analysis has also uncovered a “BRCAness” mutational signature in LMS, which confers sensitivity to DNA double‐strand break‐inducing drugs (Helleday, 2011; Chudasama *et al*, 2018) and sensitivity toward the combination of the poly(ADP‐ribose) polymerase (PARP) inhibitor olaparib and cisplatin (Chudasama *et al*, 2018). Olaparib combined with trabectedin (an alkylating drug) showed manageable toxicities at active dose levels and encouraging anti‐tumor activity in STS (Grignani *et al*, 2018). A phase 2 study on this topic is ongoing (ClinicalTrials.gov No. NCT04076579).

Exome sequencing has revealed a combination of single‐base substitutions, loss of heterozygosity events, and/or large‐scale genome instability involving 14 driver genes (*ATM*, *ATRX*, *BAP1*, *BRCA2*, *FANCA*, *MDC1, MUTYH*, *NUMA1, PTEN*, *RB1*, *RECQL4, RET*, *TP53,* and *WRN*) and many additional genes that define a “BRCAness” signature in > 80% of OS (Kovac *et al*, 2015). In fact, OS is characterized by a very complex altered genomic landscape explained by chromothripsis‐generating driver mutations and multiple genomic rearrangements (Behjati *et al*, 2017). However, in some cases, OS tumorigenesis is associated with germline alterations in *TP53*, *RB1*, and *RECQL1/2/3* predisposing patients to the accumulation of high numbers of somatic mutations (Smida *et al*, 2017; Baumhoer *et al*, 2019; Sayles *et al*, 2019). In addition, two recent publications hypothesized that specific somatic CNV profiles of OS can be used for outcome prediction and for identification of altered genes and associated pathways as potential therapeutic targets (Smida *et al*, 2017; Sayles *et al*, 2019). Similar preliminary findings have been reported for EwS and RMS (Cheng *et al*, 2019). Olaparib combined with ceritinib (ALK inhibitor) in OS showed limited toxicity and should be further evaluated (Beck *et al*, 2020). A clinical trial assessing olaparib combined with ceralasertib (ATR inhibitor) is currently in progress in the context of OS (ClinicalTrials.gov No. NCT04417062).

In contrast to OS and most sarcomas of adulthood, translocation‐driven pediatric sarcomas, such as EwS, SS, or fusion‐positive ARMS, exhibit much lower rates of single‐nucleotide variants and CNVs, and, instead, appear to be driven by marked epigenetic and transcriptomic perturbations induced by the fusion oncoproteins (Shern *et al*, 2014; Tirode *et al*, 2014; Cancer Genome Atlas Research Network, 2017). In fact, through the integration of transcriptomic and genetic data, a recent study found that EWSR1‐FLI1 hijacks the developmental transcription factor SOX6 and thus promotes proliferation of EwS cells, which provides opportunities for targeted therapeutic intervention for the oxidative stress inducer elesclomol (Marchetto *et al*, 2020). New molecular studies have also shed light on the role of the interplay between germline variants and somatic mutations in interindividual tumor heterogeneity in EwS (Musa & Grünewald, 2020). Musa *et al* recently showed that EWSR1‐FLI1 binds to a polymorphic enhancer‐like GGAA‐microsatellite, through which it regulates the expression of the oncogenic transcription factor *MYBL2* (Musa *et al*, 2019). Importantly, variability at this *MYBL2*‐associated GGAA‐microsatellite is inherited via the germline and linked to intertumoral variation in *MYBL2* expression (Musa *et al*, 2019). As MYBL2 is phosphorylated and activated by CDK2 (Musa *et al*, 2017), high MYBL2 expression sensitizes EwS cells to CDK2 inhibition, indicating the potential for using MYBL2 as a biomarker in anti‐CDK2 therapy (Musa *et al*, 2019).

While oncogenic gene fusions involving transcription factors remain largely undruggable (Knott *et al*, 2019), clinical trials using larotrectinib, a kinase inhibitor targeting gene fusions involving *NTRK1*/2/3, have shown promising results and could offer a strategy for the treatment of *NTRK*‐fusion‐positive sarcomas (Doebele *et al*, 2015; Fig 3). In addition, DNA minor groove‐binding agents in DNA, such as trabectedin or mithramycin, have been described as potent inhibitors of EWSR1‐FLI1‐mediated transcription with anti‐tumor potential (Bailey *et al*, 2019; Harlow *et al*, 2019). A recent clinical trial showed that mithramycin was too toxic at the dose required to inhibit EWSR1‐FLI1 (Grohar *et al*, 2017). However, the development of less toxic second‐generation mithramycin analogs, such as EC‐8042, opens the possibility of using this compound clinically (Osgood *et al*, 2016; Tornin *et al*, 2016; Fig 3).

### Epigenetic alterations

Mutations in chromatin remodeler components have recently been recognized as oncogenic drivers in adult and pediatric sarcomas (Nacev *et al*, 2019). Recurrent somatic missense mutations in histone H3 at lysine 36 impair the mesenchymal differentiation program and promote the initiation of UPS (Fang *et al*, 2016; Lu *et al*, 2016). These mutations result in hypomethylation of H3K36 and a gain in H3K27 methylation that leads to the de‐repression and redistribution of polycomb repressive complex 1 (PRC1) associated with a blockade of mesenchymal differentiation. K36M mutations in *H3F3B* have also been detected in most chondroblastomas (Behjati *et al*, 2013). The detection of histone mutations could help in therapeutic choices as recently evidenced by an instructive case of a patient diagnosed with a histiocytic neoplasm harboring a histone *H3K36I* mutation. This patient did not respond to multiple histiocytosis treatments, but showed a stable therapeutic response after chemotherapy and radiation therapy used for STS (Snuderl *et al*, 2019). Similarly, mutations in chromatin remodeling genes, including *ATRX*, *DOT1L*, and *H3F3A*, have been identified in 14 UPS cases highlighting the potential involvement of deregulated chromatin remodeling pathways in tumorigenesis (Ali *et al*, 2019).

Epigenetic alterations and signatures have also been extensively explored in EwS. In fact, EwS has been defined as an “enhancer disease” with substantial levels of epigenetic heterogeneity (Tomazou *et al*, 2015; Sheffield *et al*, 2017). In contrast to many other cancers, inter‐tumor epigenetic heterogeneity did not uncover discrete subgroups in EwS, but, rather, defined a continuous spectrum along two distinct and biologically interpretable dimensions (“Ewing‐like” and “mesenchymal versus stem‐like”; Sheffield *et al*, 2017). Although the clinical relevance of this epigenetic heterogeneity in sarcoma remains to be clarified, recent studies have highlighted the potential of epigenetic therapies in OS and EwS: Selective inhibition of BET bromodomain epigenetic signaling interferes with the bone‐associated tumor's vicious cycle in OS and inhibits the oncogenic transcription factor EWSR1‐FLI1 in EwS (Lamoureux *et al*, 2014; Jacques *et al*, 2016; Baud'huin *et al*, 2017). Super‐enhancers (SEs), which are large genomic regions enriched in active enhancers, have been identified as regulators of cellular identity (Whyte *et al*, 2013). In pediatric fusion‐positive ARMS, PAX3‐FOXO1 was shown to establish a miswired myoblastic SE landscape, creating a dependency on BET bromodomains (Gryder *et al*, 2017, 2019, 2020). BET inhibitors ablate PAX3‐FOXO1 function, providing a rationale for their use in the treatment of fusion‐positive ARMS patients (Gryder *et al*, 2017, 2019, 2020).

Deregulation of epigenetic programs also plays key roles in other sarcoma subtypes, such as SS, an STS that often occurs in young adults. The defining genetic event present in all histological variants of SS is the translocation of the *SS18* gene on chromosome 18q11 to an *SSX* gene (mainly *SSX1* or *SSX2*) located on chrXp11 (Clark *et al*, 1994). A recent RNA interference screen to find specific epigenetic vulnerabilities created by the SS18‐SSX oncoprotein identified a critical role for KDM2B, a member of the non‐canonical polycomb repressive complex 1 (PRC1.1) in sustaining SS cell proliferation (Banito *et al*, 2018). PRC1.1 is required for the recruitment of SS18‐SSX and the mSWI/SNF complex to unmethylated *CpG* islands, which enables the fusion to activate genes that would otherwise be repressed (Banito *et al*, 2018). In addition, two recent studies found a dependency of SS on the mSWI/SNF subunit BRD9 (Brien *et al*, 2018; Michel *et al*, 2018). However, further work should determine whether these results pinpoint a requirement of BRD9 for the SS18‐SSX‐driven expression program (Brien *et al*, 2018) and whether this constitutes a synthetic lethal interaction by regulation of fusion‐independent genomic sites (Michel *et al*, 2018).

Apart from their roles in sarcomagenesis, specific epigenetic alterations can be used to improve bone sarcoma and STS classification, diagnosis, and patient stratification (Fig 1; Koelsche *et al*, 2018a; Weidema *et al*, 2020). The promising results of brain tumor DNA methylation‐based classification (Capper *et al*, 2018) fostered adaptation of this principle to the decision‐making process in sarcoma diagnostics, which is often clinically equally challenging (Koelsche *et al*, 2018a). Analyses of more than 1,000 mesenchymal tumor samples comprising more than 50 STS and bone sarcoma subtypes of pediatric and adult patients by array‐based methylation profiling suggested that methylation signatures can be used to accurately predict sarcoma entities such as “small round blue” cell tumors (Koelsche *et al*, 2018a). Furthermore, this allows for defining novel subgroups within the sarcoma subtypes, for example, in angiosarcoma (Weidema *et al*, 2020). Methylation profiling also provides evidence for defining novel entities, such as the recently described primary intracranial sarcoma subtype with highly recurrent DICER1 mutations (Koelsche *et al*, 2018b). Thus, array‐based DNA methylation analysis will be a major step forward to quickly and reliably discriminate between mesenchymal tumor subtypes, thus increasing diagnostic accuracy. A free access classifier tool currently under development will allow sarcoma subtypes to be predicted using array‐generated DNA methylation data (www.molecularsarcomapathology.org). These molecular signatures will continue to improve the knowledge and classification of mesenchymal tumors, as well as patient outcome through more personalized therapies.

## Recent developments in functional assessment of sarcoma biology through imaging

Imaging plays a critical role in the diagnosis, staging, and monitoring of therapeutic response in sarcomas as well as in assessment of recurrence. Routine imaging modalities include plain radiography; despite limitations in contrast resolution, this modality is low cost, widely available, and useful in detecting mineralization and distinguishing ossification from calcification for diagnostic purposes (Kransdorf & Meis, 1993). Computed tomography (CT) is of limited utility in evaluating STSs due to radiation concerns and poor contrast resolution, but the ability to provide three‐dimensional information is mainly exploited to guide biopsy procedures and detect lung metastases (Casali *et al*, 2018). Magnetic resonance imaging (MRI) is the modality of choice for evaluating sarcomas, given its excellent tissue contrast and lack of ionizing radiations, particularly to determine tumor size and delineation of mass extent and to identify invasion of the compartments and occasionally for histological classification using conventional T_1_‐weighted, T_2_‐weighted, and fluid‐sensitive sequences (Fayad *et al*, 2012).

In addition to these common imaging modalities, novel techniques are emerging for the functional characterization of tumors, including metabolism and the microenvironment, and for a reliable estimation of treatment response by complementing functional assessments with anatomical evaluation. PET, in combination with ^18^F 2‐fluoro‐2‐deoxy‐D‐glucose (FDG), is a valuable tool for the characterization of cancer metabolism, since the uptake of FDG—a non‐metabolizable derivative of native glucose—correlates with the pathological grade and can be used to discriminate between benign lesions and STSs (Ioannidis & Lau, 2003). Moreover, it can be used to detect metastases for the follow‐up of treatments and to identify the target regions for biopsy (Kubo *et al*, 2016; Harrison *et al*, 2017).

Magnetic resonance imaging has taken a lead in the functional characterization of tumors, since it has the capability to provide multiparametric analysis of biological features of sarcoma by exploiting a variety of approaches, including chemical shift imaging (CSI), diffusion‐weighted imaging (DWI), magnetic resonance spectroscopy (MRS), and quantitative dynamic contrast‐enhanced (DCE)‐MRI (Subhawong & Wilky, 2015). DCE‐MRI provides information on tissue vascularization, perfusion, and permeability that can be exploited for differentiating STS from benign soft tissue tumors (Tuncbilek *et al*, 2005; Pepin *et al*, in press), or in monitoring tumor response by revealing early perfusion changes (Amit *et al*, 2014; Crombé *et al*, 2019), or in cell proliferation assessment (Lee *et al*, 2020). DWI provides measurements of tissue cellularity and membrane integrity by assessing the Brownian motion of water molecules in tissues. Malignant lesions are usually more cellular than benign lesions, leading to modified Brownian motion (Amit *et al*, 2014). DWI may be particularly suited for assessing treatment response, with an increase in water diffusion that is usually associated with a positive therapeutic response (Dudeck *et al*, 2008). MRS can provide the metabolic profile of tumors and is frequently used in sarcoma to evaluate the concentration of the membrane phospholipid choline, which may serve as a marker of malignancy in musculoskeletal STSs (Fayad *et al*, 2007, 2012). The quantitative parameters of CSI, DWI, MRS, and DCE‐MRI have also shown promising potential as biomarkers for osseous tumors (e.g., differentiation of tumor from edema, determination of biological aggressiveness) (Fukuda *et al*, 2019).

Tumor acidosis is considered a major player in promoting tumor angiogenesis, progression, invasion, and resistance to chemo‐radiotherapy (Pillai *et al*, 2019). In OS, the acidic microenvironment strongly affects the activation of MSCs by inducing clonogenicity and invasion, in addition to promoting multidrug resistance (described above) (Avnet *et al*, 2016, 2017). Indirect measurements of acidic regions in the TME have been obtained in canine OS samples by immunohistochemistry (IHC) analysis (Avnet *et al*, 2017). Consequently, non‐invasive imaging approaches are needed to provide accurate *in vivo* measurements of tumor acidosis (Anemone *et al*, 2019; Consolino *et al*, 2020). Previous MRS approaches reported intratumoral acidosis in murine fibrosarcoma models, but lacked the ability to assess the spatial distribution (Vaupel *et al*, 1989, 1994). Recently, a novel MRI‐based approach has been proposed for *in vivo* imaging of extracellular tumor pH with high accuracy and spatial resolution by exploiting iopamidol, an FDA‐approved X‐ray contrast medium that allows potential clinical translation (Longo *et al*, 2014; Anemone *et al*, 2019). Preclinical studies have shown the capability of this pH mapping method to assess the correlation between dysregulated glycolysis and tumor acidosis (Longo *et al*, 2016) and monitor the treatment response to anticancer therapies targeting glycolysis (Anemone *et al*, 2017). This novel tumor pH imaging approach may be of particular importance for investigating tumor acidosis in the field of sarcomas.

It is interesting to note that advances in imaging technology have paved the way for imaging modalities that are capable of defining drug response at earlier stages of treatment. As an example, the use of FDG‐PET after 2 weeks of treatment with pazopanib was able to correctly classify 42% of STS patients as non‐responders (Vlenterie *et al*, 2019).

## Novel biomarkers of sarcomas

Traditionally, histomorphological assessment of sarcoma samples in conjunction with clinical and imaging features (See section “Recent developments in functional assessment of sarcoma biology through imaging”) has led to the establishment of diagnosis. In addition, the identification of fusion gene products or overexpressed oncogenes by IHC has enriched the clinical practice (Heymann, 2014; WHO Classification of Tumours: Soft Tissue and Bone Tumours, 2020). However, sarcomas often do not express specific IHC markers. In contrast to studies on tumor biopsies, the discovery of circulating tumor cells (CTCs), cell‐free circulating tumor DNA (cfDNA), and tumor‐derived extracellular vesicles (EVs), as well as the advent of new technologies to detect, quantify, and analyze these biological entities in peripheral blood, hold great promise for developing minimally invasive methods to improve patient care. Indeed, liquid biopsies may enable longitudinal monitoring of treatment response, early detection of relapse, and the identification of druggable driver mutations. Although IHC markers remain important tools for diagnostics in sarcomas (as reviewed in ref. Wei *et al*, 2017), the aim of this section is to focus on recent advances in the field of liquid biopsies in sarcoma.

### Circulating cytokines as markers associated with prognosis

Deregulated levels of cytokines and their receptors can be detected in cancer patients both locally and systemically, and they may be of a high prognostic value in several tumor types (Kumar *et al*, 1998; Belluco *et al*, 2000; Kawashima *et al*, 2000), including sarcomas. Increased serum levels of cytokines and their soluble receptors that are involved in bone degradation (e.g., IL‐6 and IL‐8) and bone formation (e.g., tumor necrosis factor receptor I [TNFRI]) are positively correlated with tumor size and local tumor extent, which is associated with worse overall survival in adult bone sarcoma patients (Rutkowski *et al*, 2003). Several studies have recognized the negative prognostic significance of various chemokines or cytokines, such as CXCL4/CXCL6 (Li *et al*, 2011), CXCL10 (Flores *et al*, 2017), IL‐17A (Wang *et al*, 2013), IL‐6, IL‐8, and TNF‐α (Xiao *et al*, 2014) in OS patients. IL‐6 levels were also elevated in serum of a subgroup of EwS patients with poor prognosis (Lissat *et al*, 2015) and constitute an indicator of poor overall survival and event‐free survival in STS, suggesting a possible association with aggressive tumor behavior (Hagi *et al*, 2017). Besides IL‐6, other cytokine signaling components including IL‐8, TNF‐R, sIL‐2R, and M‐CSF have been shown to correlate with tumor grade and size in STS patients, and the serum levels of some of these proteins were associated with the prognosis (Rutkowski *et al*, 2003). To date, the identification of specific cytokine components involved in sarcoma progression is far from being complete, and future studies are essential for generating innovative prognostic tools and facilitating therapy and risk‐stratification.

### Extracellular vesicles (EVs) and micro RNAs (miRNAs)

EVs are intercellular messengers where cargo (nucleic acids, proteins, lipids, and metabolites) can be characterized and potentially used as new or supplementary biomarkers in liquid biopsy approaches (Mader & Pantel, 2017). EVs isolated from peripheral blood samples derive not only from tumor cells but also from cells of the TME (See section “The complex biology of sarcoma: How current knowledge may affect therapy”). Thus, EVs can be representative of the interaction between cells in the TME and may bring useful information to follow disease progression (Baglio *et al*, 2017; Mannerström *et al*, 2019). One major advantage of EVs in the liquid biopsy approaches is their membranous structure that protects their cargo and gives them enough stability to allow EV sample storage before analysis, which facilitates their clinical use (Jeyaram & Jay, 2017).

In 2013, Miller *et al* initiated the study of EVs’ diagnostic potential for sarcoma by demonstrating the efficient isolation of EVs derived from EwS and containing EwS‐specific transcripts, including *EWSR1*‐*FLI1,* in a pre‐clinical model for patient plasma (Miller *et al*, 2013). Since then, only few clinical studies have been conducted in limited patient cohorts exploring sarcoma‐derived EVs as biomarkers. Circulating EV‐associated transforming growth factor β (TGF‐β) levels were elevated in OS patients compared with healthy individuals (Baglio *et al*, 2017), and circulating vesicular *miR‐25-3p* and *miR‐92a-3p* were elevated in LPS patients (Casadei *et al*, 2017). Moreover, *miR‐25-3p* and *miR‐92a-3p* modulated macrophages in the local TME, which in turn released IL‐6, increasing the proliferation, migration, and invasion of cancer cells. EVs secreted by dedifferentiated LPSs were also carriers of *MDM2* DNA transferable to preadipocytes, which acquired oncogenic properties (e.g., impaired *TP53*) (Casadei *et al*, 2019). In addition, *miR‐642a*, *miR‐1260b,* and *miR‐4286* were significantly higher in serum collected from myxofibrosarcoma patients compared with healthy controls, and *miR‐1260b* expression was associated with tumor burden and the infiltrative nature of sarcoma (Morita *et al*, 2020). Moreover, EVs derived from the plasma of GIST patients expressed activated KIT, which was undetectable in samples from healthy donors (Atay & Godwin, 2014). Promising data were also obtained for SS, where serum *miR‐92b-3p* constituted a robust marker for discriminating patients with SS from other STS patients and was elevated in EVs compared with *AGO2*‐positive fractions (Uotani *et al*, 2017). *miR‐761* released in EVs enhanced pazopanib resistance in SS (Shiozawa *et al*, 2018) and correlated with increased resistance. Such resistance may be explained by the modulation of NAD‐dependent protein deacetylase sirtuin‐3 (SIRT3) expression. Interestingly, pazopanib regulated the protein contents of EVs released by SS (Shiozawa *et al*, 2018), more specifically proteins from the Wnt pathway, which is crucial for SS (Baird *et al*, 2005). RMS also secreted EVs, which upregulated the proliferation of RMS cells and fibroblasts of the TME, and initiated the migration/invasion of tumor‐associated fibroblasts through promotion of angiogenesis (Ghayad *et al*, 2016). EVs secreted by cancer cells appeared as key regulators of bone sarcoma biology. A pilot study analyzing RNA isolated from plasma‐derived EVs of OS patients found a higher tumor mutational burden in patients with metastatic disease than in OS patients without metastases (Bao *et al*, 2018). The response to chemotherapy can be monitored by the identification of dysregulated levels of miRNAs (*miR‐124*, *miR‐133a*, *miR‐135b*, *miR‐148a*, *miR‐199a-3p*, *miR‐27a*, *miR‐385*, and *miR‐9*) and mRNAs (*ANNEXIN2*, *CDC5L*, *CDKN1B*, *CIP4*, *MTAP*, *PEDF*, *SMAD2*, and *WWOX*) in EVs isolated from the serum of OS patients with a poor chemotherapeutic response when compared with good responders (Xu *et al*, 2017). However, before being incorporated into routine clinical practice, a careful optimization and standardization of EVs isolation protocols from blood samples and validation studies in larger patient cohorts are required. In particular, the position paper recently published by the International Society for Extracellular Vesicles stresses the importance of a variety of critical parameters (pre‐analytical parameters, such as time to processing, type of container(s), and choice of anticoagulant) (Théry *et al*, 2018).

### Circulating tumor cells (CTCs)

Circulating tumor cells are cells released from primary and metastatic tumor foci and migrating in secondary organs through the peripheral blood. The biological value of CTCs was assessed by comparing the molecular profiles of CTCs and primary tumors (Keller & Pantel, 2019). Controversial conclusions showed that CTCs only partly reflect the spectrum of mutations in the primary and metastatic tumors (Paoletti *et al*, 2018; Wu *et al*, 2018; Brown *et al*, 2019; Keller & Pantel, 2019). CTCs may be considered a snapshot of tumor tissue heterogeneity at a given time and could have strong implications for longitudinal patient monitoring (Brown *et al*, 2019; Tellez‐Gabriel *et al*, 2019). In contrast to studies in carcinomas (Pantel & Alix‐Panabières, 2019), studies of CTCs in sarcomas are currently limited (Tellez‐Gabriel *et al*, 2016). The restricted number of patients, the high heterogeneity of sarcoma subtypes, and the absence of specific markers expressed by most sarcoma cells contribute to the limited advances in this field. Despite the absence of specific markers, various methods of cell isolation based on physical specificity (e.g., higher size and higher cell deformability of tumor cells) or biological properties (e.g., immunomagnetic isolation) have been proposed with success (Gabriel *et al*, 2016; Hayashi *et al*, 2017; Li *et al*, 2017). CTCs are detectable in bone sarcomas (Chinen *et al*, 2014; Benini *et al*, 2018) and STS patients (Braun *et al*, 2018; Mihály *et al*, 2018; Przybyl *et al*, 2019). To improve the sensitivity and specificity of detection and isolation of CTCs across sarcoma subtypes, investigators have been looking for universal sarcoma markers (Satelli *et al*, 2014; Li *et al*, 2018). Cell‐surface Vimentin was expressed in CTCs isolated from 24 sarcoma patients comprising OS, EwS, angiosarcoma, LMS, and UPS (Satelli *et al*, 2014). More recently, a new class of CD45^−^ CTCs expressing macrophage markers CD14 and CD68, cell‐surface Vimentin, and specific GIST markers (DOG1 and KIT) have been identified (Li *et al*, 2018). This CTC subset was more abundant in patients with metastatic disease than with localized GIST. In contrast, cell‐surface Vimentin‐positive cells that did not express macrophage markers failed to predict GIST metastasis (Li *et al*, 2018). These studies underlined the potential clinical interest in CTCs as prognostic or predictive markers, although longitudinal clinical trials with a large series of patients may be required.

### Cell‐free circulating tumor DNA (cfDNA)

cfDNA is composed of DNA fragments released into the bloodstream by healthy and cancer tissues alike, as a result of cell death (e.g., apoptosis, necrosis) or active release (Volckmar *et al*, 2018; Chen & Zhao, 2019). The cfDNA fraction released from tumor tissues, called circulating tumor DNA (ctDNA), may reflect the genetic aberrations of cancer cells at a given time. cfDNA was recently detected in plasma of bone sarcoma (Gutteridge *et al*, 2017; Shukla *et al*, 2017; Barris *et al*, 2018) and STS patients (Boonstra *et al*, 2018; Eastley *et al*, 2018; Namløs *et al*, 2018; Ogino *et al*, 2018; Shulman *et al*, 2018). In these studies, total cfDNA levels were frequently increased in the plasma of sarcoma patients compared with the cancer‐free controls. Cancer‐associated mutations, such as in *TP53*, *PIK3CA*, and *IDH1* or fusion oncogenes (e.g., *SS18‐SSX1/2*), were also detected. In patients affected by GIST, mutations of *KIT* and *PDGFRA* were detected, and the amount of mutant cfDNA correlated with clinical progression (Maier *et al*, 2013). Interestingly, the usefulness of cfDNA analysis was demonstrated to identify TKI‐resistant mutations (Yoo *et al*, 2014). In a series of CHSs, ctDNA levels detected by mutated *IDH1* correlated with tumor grade and prognosis (Gutteridge *et al*, 2017). Patient‐specific somatic alterations in cfDNA were observed in OS (Barris *et al*, 2018) and were associated with inferior outcomes in EwS and OS patients (Shulman *et al*, 2018). Individual genomic *EWSR1‐ETS* fusion sequences can be quantified from cfDNA in EwS patients’ plasma, and as such represent suitable serum markers for therapy assessment (Krumbholz *et al*, 2016). Indeed, copy numbers of cell‐free *EWSR1‐ETS* fusion sequences correlate with patients’ risk factors such as tumor volume, pelvic tumor, and metastatic status, and most EwS patients show a fast reduction of cfDNA levels during treatment, while recurrence of increasing cfDNA levels indicates relapse (Krumbholz *et al*, 2016). In addition to somatic mutations and DNA methylation, recent studies have reported the detection of circulating nucleosomes in blood, showing that cfDNA retains at least some features of nuclear chromatin. Most importantly, whole‐genome sequencing of cfDNA was shown to yield a dense, genome‐wide map of nucleosome occupancy that enables identification of the cell types that contribute to circulating cfDNA (Snyder *et al*, 2016; Ulz *et al*, 2016). This is highly relevant to EwS as it supports the idea of monitoring the chromatin state of EwS‐specific enhancer elements (Riggi *et al*, 2014; Tomazou *et al*, 2015; Sheffield *et al*, 2017) over time and during the treatment course, enabling the development of enhancer‐based minimally invasive assays for live monitoring of therapy response.

Overall, the detection and characterization of cfDNA and ctDNA in sarcomas show promising results, and efforts are now needed to profile larger biological cohorts with complete clinical annotations to validate their clinical value.

## Recent therapeutic developments

### Precision medicine in sarcoma: General considerations

The ultimate goal of personalized medicine is to be able to integrate clinical, genomic, transcriptomic, and epigenomic data to increase the accuracy of diagnosis and prognosis, and to identify the most effective therapy for treatment (Burdach *et al*, 2018; Salgado *et al*, 2018; Gargallo *et al*, 2020). Recent advances in machine learning‐based methods for analysis of histology and radiography imaging may also play an increasingly important role (Blackledge *et al*, 2019; Wang *et al*, 2019; Malinauskaite *et al*, 2020). For instance, clinical investigations into immune checkpoint therapy have designated UPS, myxofibrosarcoma, and similar genomically complex histotypes as “UPS” (Que *et al*, 2017), making comparisons with other studies difficult. However, the inclusion of genomic analyses led to the re‐classification of 13% of sarcoma cases and would have resulted in changes to the clinical treatment pathway or prognosis in 11% of cases, demonstrating the importance of including molecular and computational tools for classification and risk‐stratification of sarcomas (Italiano *et al*, 2016).

Several recent studies have identified therapeutically targetable mutations in sarcoma patients and have used this knowledge to guide treatment (Groisberg *et al*, 2017). Yet, not all attempts were successful (Demetri *et al*, 2013; Perry *et al*, 2014), indicating that genomic data alone are not sufficient for the accurate prediction of response to therapy.

The clinical trial MULTISARC (ClinicalTrials.gov No. NCT03784014) should provide the first glimpse into the successes and potential pitfalls of personalized medicine in sarcoma. Based on a retrospective survey of genomic alterations that could be therapeutically actionable (Lucchesi *et al*, 2018), MULTISARC is a two‐arm, randomized trial aiming to prospectively evaluate their potential as predicative biomarkers for response to therapy. STS patients will be randomized to receive standard therapy or undergo genomic profiling for suitability for therapy with 16 different agents. Sarcomas were identified as a priority for the 100,000 genomes project in the United Kingdom with 500 to be sequenced as part of the study, although it will focus on LMS, myxofibrosarcoma, SS, and rare histotypes such as alveolar soft part sarcoma (ASPS). In addition to collecting both genomic and clinical data from patients, the project's Genomics England Clinical Interpretation Partnerships (GeCIPs), including the Sarcoma GeCIP, will also identify training and standardization of practice needed to bring personalized medicine toward routine clinical practice.

Likewise, genomic analyses in combination with screening cancer cell lines against libraries of drugs have the potential to improve the correlation between genomic biomarkers and response to therapy. Such an approach has been used to identify biomarkers for response to therapy of several sarcomas using cell lines, patient‐derived samples, and canine sarcoma as proof of principle (Berlow *et al*, 2019). This approach is challenging for studying sarcoma, due to the limited number of cell lines available, although isolation of new cell lines (Salawu *et al*, 2016) and sarcoma PDX models is improving (Stebbing *et al*, 2014). The next step will be to take advantage of combining molecular information gained through next‐generation sequencing (NGS) technologies with functional drug screening using primary organoid cultures that include both stromal cells and cancerous cells to improve prediction of response to therapy, as observed in other cancers (Tiriac *et al*, 2018; Vlachogiannis *et al*, 2018).

### Photodynamic therapy

An interesting approach, based on photo‐ and radiodynamic therapy following acridine orange administration, has been extensively investigated and successfully applied for the treatment of sarcomas (Matsubara *et al*, 2013; Kusuzaki *et al*, 2018; Martano *et al*, 2019). Photodynamic therapy with hematoporphyrin prevented local recurrence following minimally invasive surgery in preclinical models (Duchi *et al*, 2016) as well as in clinical settings (Hourigan *et al*, 1993). Acridine orange has the advantage of selectively binding to tumor tissue due to the acidic microenvironment specific to malignant cells (Matsubara *et al*, 2006) and to specifically exert a strong cytotoxic activity on tumor cells, which is further enhanced by photo‐ and radioactivation (Matsubara *et al*, 2013; Kusuzaki *et al*, 2018). Therefore, following marginal or even intralesional gross removal of the tumor, it is possible to selectively target residual sarcoma and spare the surrounding normal tissues, with a satisfactory functional result (Martano *et al*, 2019). The procedure is safe, without local or systemic complications (Martano *et al*, 2019). Systemic administration of acridine orange with low‐dose radiation therapy is currently under evaluation in Japan for non‐resectable sarcomas (Kusuzaki *et al*, 2018). This procedure appears to be safe, and the preliminary results are encouraging.

### Immune‐based therapies

Sarcomas are highly heterogeneous, including the TME, which might dictate their heterogeneous response to different immunotherapeutic approaches (section “The complex biology of sarcoma: How current knowledge may affect therapy”, Figs 2 and 3). While checkpoint inhibitor immunotherapies have already been introduced for the first‐/second‐line treatment of several carcinomas, their efficacy in sarcoma treatment is currently unclear, and clinical trials are ongoing (Thanindratarn *et al*, 2019). Unfortunately, the first results showed only sporadic therapeutic responses in STSs and bone sarcomas, highlighting the need for further investigations (Merchant *et al*, 2016).

Some STS subtypes (e.g., myxofibrosarcoma and UPS) are characterized by a high mutational burden, which may constitute a biomarker for response to immune checkpoint blockade (Pollack *et al*, 2017). In addition, recent profiling studies of immune checkpoints expression in STSs and bone sarcomas revealed their correlation with poor clinical outcomes and provide rationales for their targeting (Dancsok *et al*, 2019; Orth *et al*, 2020). In fact, a new study revealed a positive correlation between immune infiltration and response to anti‐PD‐L1 therapy in sarcoma (Keung *et al*, 2020). Similarly, a gene expression study in 608 tumors across STS subtypes established a classification between immune‐low, immune‐high, and vascularized phenotypes (Petitprez *et al*, 2020). The phenotype with the highest immune cell infiltration featured tertiary lymphoid structures with T cells, dendritic cells, and B cells. Interestingly, B cells were the strongest prognostic factor, and they were associated with improved survival and high response rates to PD‐1 blockade (Petitprez *et al*, 2020).

Therapeutic strategies based on (genetically modified) T cells are currently underway. Their main objectives are to enhance T‐cell infiltration into tumor tissues and identify specific tumor target antigens only expressed by malignant cells (Baldauf *et al*, 2018b). Some encouraging results have been described, such as the therapeutic benefit observed in SS upon inoculation of autologous T cells engineered to express an affinity‐enhanced T‐cell receptor (TCR) recognizing the NY‐ESO‐1‐derived peptide (D'Angelo *et al*, 2018). Similarly, chimeric antigen receptor (CAR) T cells characterized by the expression of a chimeric receptor (fusion of specific antibody‐derived single‐chain variable fragments with the signaling domain of a T‐cell receptor) are capable of inducing conventional activation signals from TCRs in a non‐MHC restricted manner (Majzner & Mackall, 2018; Pollack *et al*, 2018). Although some sarcomas subtypes express tumor epitopes, such as HER2, GD2, ROR2, or EGFRvIII, B7‐H3 (Majzner *et al*, 2019), or oncofetal glycosaminoglycans (Salanti *et al*, 2015), these tumor epitopes are often only expressed at low levels. CAR T cells may overcome the low levels of tumor antigen expression, and several clinical trials are currently in progress to evaluate their therapeutic benefit (Majzner & Mackall, 2018; Pollack *et al*, 2018). Interestingly, a first completed phase I/II trial with HER2‐CAR T cells showed that cells can persist for 6 weeks without evident toxicities, setting the stage for studies that combine CAR T cells with other immunomodulatory approaches to enhance their expansion and persistence (ClinicalTrials.gov No. NCT00902044; Ahmed *et al*, 2015). OS (Théoleyre *et al*, 2005; Koirala *et al*, 2016), EwS (Machado *et al*, 2018), and CHS (Simard *et al*, 2017; Richert *et al*, 2019) are moderately infiltrated by lymphocytes with moderate functional impact (Heymann *et al*, in press). However, the number of T lymphocytes appeared to be significantly higher in metastatic foci than in primary tumors and in local relapses, suggesting the potential benefit of TIL‐based immunotherapy in metastatic clinical situation (Sundara *et al*, 2017; Shi *et al*, 2020). T lymphocyte infiltration has also been described in STS (Dancsok *et al*, 2019; Que *et al*, 2019; Shi *et al*, 2020). Two phase 2 clinical trials have recently been set up for treating sarcoma patients with autologous TIL expanded *ex vivo* (ClinicalTrials.gov No. NCT03449108 & NCT03935893). Similarly, adoptive immune cell therapy options based on infusion of NK cells were assessed in preclinical models of bone sarcomas and STS (Thiel *et al*, 2013; Fernández *et al*, 2015). Case reports including ERMS and EwS showed a beneficial anti‐tumor activity of allogeneic hematopoietic stem cell transplantation (Pérez‐Martínez *et al*, 2009). A pilot phase 1/2 clinical study named “NKEXPSARC” will assess the clinical potential of activated haploidentical natural killer cell infusions in sarcomas (ClinicalTrial.gov No. NCT02409576).

### Oncolytic viruses

The approval of the Herpes virus *Talimogene Laherparepvec* (T‐VEC; Imlygic) by the FDA and EMA for recurrent melanoma confirms that virotherapy has emerged as a feasible therapeutic strategy in oncology (Andtbacka *et al*, 2015; Ribas *et al*, 2017). Oncolytic viruses have been assessed in bone sarcomas and STSs (Lacroix *et al*, 2018; MacNeill *et al*, 2018; Smith *et al*, 2019; Tazawa *et al*, 2020). They are tumor selective, destroy cancer cells, and trigger an anti‐tumor immune response (Garcia‐Moure *et al*, 2017; Varela‐Guruceaga *et al*, 2018). Table 2 summarizes the main potential therapeutic viruses for the treatment of sarcomas.

**Table 2:** Summary of main oncolytic viruses applied in sarcoma treatment

Virus	Disease	Trial
DNA		
Adenovirus (Ad)	Respiratory and gastrointestinal infections	Preclinicalphase I
Herpes simplex virus (HSV)	Oral and genital ulcerations	Preclinicalphase I
Vaccinia virus	Flu	Preclinical
RNA		
Reovirus	Respiratory and gastrointestinal infections	Preclinicalphase I
Semliki forest virus (SFV)	Non‐pathogenic in humans / encephalitis in mice	Preclinical
Vesicular stomatitis virus (VSV)	Non‐pathogenic	Preclinical
Measles virus (MeV)	Measles	Preclinical
Poliovirus	Neurological disorders (poliomyelitis)	Preclinical
Newcastle disease virus (NDV	Respiratory and gastrointestinal infections	Preclinicalphase I/II

In the group of DNA viruses, *Adenovirus*, *Herpes virus,* and *Vaccinia virus* are commonly employed. These three types of viruses have advanced to clinical trials. For example, *Telomelysin*, a human telomerase reverse transcriptase (*hTERT*) promoter‐driven modified oncolytic *Adenovirus*, was tested in a phase I clinical trial to assess its clinical safety in patients with advanced solid tumors (Nemunaitis *et al*, 2010). *Herpes virus HSV1716* was tested in pediatric patients with non‐central nervous system solid tumors (ClinicalTrials.gov No. NCT00931931), including two patients with OS (Streby *et al*, 2017). This virus was delivered as a single dose of 10^5^–10^7^ infectious units via CT‐guided intratumoral injection, and tumor response was measured by imaging. *HSV1716* was safe in the pediatric population, with minimal toxicities reported; however, no clinical responses were observed in this phase I trial (Streby *et al*, 2017). Finally, the *Vaccinia virus*, armed with GM‐CSF (JX‐594), has also been tested in a phase I clinical trial in pediatric solid tumors (ClinicalTrials.gov No. NTC01169584) but did not include sarcomas. This virus did not show toxicity, but exhibited biological activity in the pediatric population (Cripe *et al*, 2015). The group of RNA viruses, including *Semliki Forest Virus*, *Poliovirus*, *Newcastle Disease Virus*, *Measles*, or *Reovirus* (Table 2), have also transitioned to clinical trials (Schneider *et al*, 2018). However, only the *Reovirus Reolysin* has been tested in OS (Kolb *et al*, 2015). Twenty‐four patients were treated in this trial, including OS and other extracranial pediatric tumors, to establish virus safety. The virus was well tolerated and showed a safe profile, but no response was observed (Kolb *et al*, 2015).

**Figure 4:**
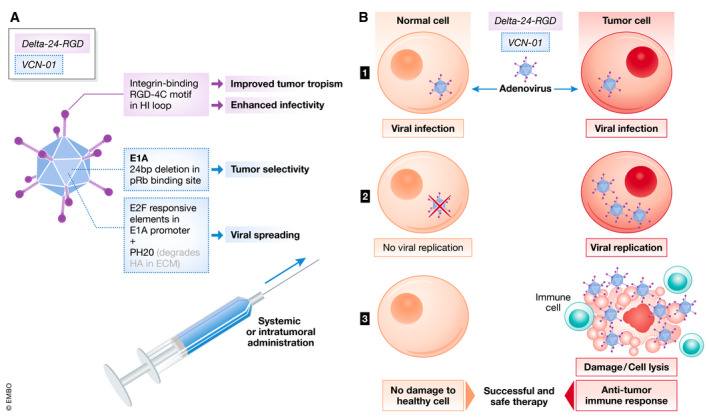
Main features and functional aspects of oncolytic virus (A) Characteristics of oncolytic *Adenoviruses Delta‐24-RGD* and *VCN‐01*. These two *Adenoviruses* harbor different modifications (black for *Delta‐24-RG* (D24‐RGD) and dashed blue for *VCN‐01*) that render them with tumor specificity and enhanced infectivity. (B) Schematic representation of the virus’ mechanism of action. (1) The viruses are able to infect both normal and tumor cells. (2) However, due to their tumor specificity they only replicate and lyse the tumor cells. (3) They exert a potent cytolytic effect, and they are able to trigger an anti‐tumor immune response, which is crucial to successfully eliminate the tumors.

The therapeutic effect of several oncolytic viruses in STSs (Leddon *et al*, 2015; Siurala *et al*, 2015; Wilkinson *et al*, 2016; Chen *et al*, 2017) and bone sarcomas (Witlox *et al*, 2004; Graat *et al*, 2006; Hingorani *et al*, 2014; Martínez‐Vélez *et al*, 2016; Martinez‐Velez *et al*, 2014) was tested in various preclinical studies. Due to their versatility and lack of toxicity, oncolytic *Adenoviruses* are commonly used (Fig 4). Because the Rb pathway is frequently mutated in sarcomas, oncolytic *Adenoviruses* based on selective replication conditional to Rb pathway deregulation have been developed. *VCN‐01* (Martínez‐Vélez *et al*, 2016) and *Delta‐24-RGD* (Martinez‐Velez *et al*, 2014) are *Adenoviruses* that showed *in vitro* and *in vivo* anti‐sarcoma activity. *Delta‐24-RGD* is a replication‐competent *Adenovirus* that harbors a 24‐bp deletion in the E1A region (responsible for binding Rb protein) that triggers tumor selectivity. The addition of an RGD‐4C motif in the fiber H1 loop allows enhanced infectivity through integrins that are widely expressed in cancer cells (Suzuki *et al*, 2001). *VCN‐01* is an oncolytic *Adenovirus* where the *E1A* gene also contains deletions in the pRb binding site, thus rendering its selective replication in *Rb*‐deficient tumor cells (Rodríguez‐García *et al*, 2015). Importantly, both viruses have shown efficacy not only against the primary tumor but also against lung metastases (Martinez‐Velez *et al*, 2014; Martínez‐Vélez *et al*, 2016). It should be noted that most of the oncolytic *Adenoviruses* are amenable to be used in combination with standard chemotherapy, small molecules, nanoparticles, immunotherapy with immune checkpoint inhibitors, and CAR T cells.

## Conclusions

Sarcomas comprise relatively rare but diverse cancer entities affecting patients of all ages. Bone sarcomas are more frequent in adolescents and young adults, and the frequency of STS increases with age. Most sarcomas exhibit a high cellular, molecular, and genetic/epigenetic heterogeneity, which makes identification of single therapeutic targets more difficult. Fortunately, in some instances, identification of new targets has revolutionized the therapeutic management of sarcoma patients, as illustrated by the use of imatinib mesylate targeting receptor tyrosine kinases in GIST even if secondary resistance is observed (Napolitano & Vincenzi, 2019), which can be overcome with other, rationally designed TKIs (Blay *et al*, 2020). The TME plays a key role in the pathogenesis of sarcomas, not only for tumor initiation but also in the metastatic process. Like other cancers, sarcomas are now in the era of immunotherapy (e.g., PD‐L1 inhibitors, CAR T‐cell therapy) and numerous clinical trials are currently ongoing. Epigenetic profiles emerge as useful tools to improve diagnostic accuracy in sarcomas and to discover or better delineate new sarcoma subtypes. In addition, epigenetic events occurring during sarcomagenesis have been identified as new, promising opportunities for treating sarcomas. Innate or acquired resistances of sarcomas are the principal obstacles to treatment efficacy, and a better understanding of these cellular/molecular processes will help to define better therapeutic lines. Tackling MDR, CSCs, and/or cell dormancy are all tracks for progress. Finally, the high heterogeneity of sarcoma requires better classification of sarcoma subtypes based on (epi)genetic characteristics (e.g., CTCs, circulating RNA/DNA, immune infiltrates) to identify the best therapeutic option for each patient. Thus, future advances in the field of molecular biology related to sarcomas hold great promise to overcome treatment resistance and treatment‐related toxicity through individualized precision medicine approaches.

## Conflict of interest

Marta Alonso has obtained research grant from DNAtrix. Stefan Budach has an ownership interest in PDL BioPharma and has had US and EU intellectual properties in gene expression analysis. He served as consultant to EOS Biotechnology Inc. and serves as advisor to Bayer AG and Swedish Orphan Biovitrum AB. Dominique Heymann has an ownership interest in Atlanta SAS (Saint‐Herblain, France).

**Pending issues**

• Identification of unknown extrinsic factors that may have a role in sarcoma progression and response to therapy and that may derive from the following: (i) the tumor microbiome, (ii) immune infiltrates, and (iii) other cells of the tumor‐associated stroma (including neurons).
• Development of novel and more representative 3D preclinical models to be used in place of animal models to develop new therapeutic options.
• Further generation of immunocompetent and *bona fide* GEMMs for all sarcoma subtypes for a better understanding of sarcomagenesis.
• Elucidation of the mechanisms that lead to resistance toward TKIs in non‐GIST STS.
• Elaboration of non‐invasive assays for the monitoring of drug response and for early detection of drug resistance.
• Development of compounds that enhance tumor antigen presentation and of therapeutic protocols based on immunotherapies for the treatment of sarcoma.
• Investigation of the use of photodynamic therapies for limb‐preserving surgery.
• Optimization and clinical translation of oncolytic virus therapies for sarcomas.

## For more information

Societies and Network for health scientists and professionals

• EuSARC (European network for SARComa): https://eusarc.com/
• NIH website for information to health professional, related to bone cancer: https://www.cancer.gov/types/bone/hp
• World sarcoma network: http://www.worldsarcomanetwork.com/

Patient associations

• UK patient association on sarcoma: https://sarcoma.org.uk/about-sarcoma/understanding-sarcoma-0
• The Liddy Shriver sarcoma initiative: http://sarcomahelp.org/sarcoma-centers.html#tpm1_1
• Sarcoma patients Euronet: https://www.sarcoma-patients.eu/it/sarcoma-research/research-networks

OMIM site

• Ewing sarcoma: https://www.omim.org/entry/612219?search=sarcoma&highlight=sarcoma
• GastroIntestinal Stromal Tumor: https://www.omim.org/entry/606764?search=GIST&highlight=gist
• Kaposi sarcoma: https://www.omim.org/entry/148000?search=sarcoma&highlight=sarcoma
• Osteosarcoma: https://www.omim.org/entry/259500?search=osteosarcoma&highlight=osteosarcoma
• Synovial sarcoma: https://www.omim.org/entry/300813?search=sarcoma&highlight=sarcoma

Database

• Surveillance, Epidemiology, and End Results (SEER) database: https://seer.cancer.gov/statfacts/html/bones.html
• National Program of Cancer Registries (NPCR): https://www.cdc.gov/cancer/npcr/index.htm
• National Cancer Database (NCDB): https://www.facs.org/quality-programs/cancer/ncdb
• ClinicalTrials.gov: https://clinicaltrials.gov/

Reference book

• WHO Classification of Tumours, 5th Edition, Volume 3. Soft Tissue and Bone Tumours WHO Classification of Tumours Editorial Board. IARC publication Ed. (Lyon, FR) 2020: https://www.iarc.fr/news-events/publication-of-the-who-classification-of-tumours-5th-edition-volume-3-soft-tissue-and-bone-tumours/

Diagnostic sarcoma classifier

• DNA methylation‐based classification: https://www.molecularsarcomapathology.org
